# When honesty and cheating pay off: the evolution of honest and dishonest equilibria in a conventional signalling game

**DOI:** 10.1186/s12862-017-1112-y

**Published:** 2017-12-28

**Authors:** Szabolcs Számadó

**Affiliations:** MTA TK “Lendület” Research Center for Educational and Network Studies (RECENS), Tóth Kálmán u. 4, Budapest, H-1097 Hungary

**Keywords:** Honest signalling, Deception, Mixed ESS, Frequency dependent selection, Aggressive communication, Animal personality

## Abstract

**Background:**

The reliability of signals is a key issue in the study of animal communication. Both empirical work and theoretical models show that communication need not be entirely honest, and thus signals can be deceitful. Aggressive communication appears to be a prime candidate for such deceitful communication, because bluffing has been described in several species. Bluffing in these situations are supposed to be maintained by frequency dependent selection where the fitness of a given type depends on the frequencies of the other types in the population. Previous efforts to model such a scenario through individual based simulations have yielded conflicting results. Studies have either found a rich set of polymorphic strategies including the traditional cheating scenario or found none. Thus, the modelling assumptions responsible for these diverging conclusions remain unclear.

**Results:**

In this study, I investigate the effects of four modelling assumptions: the role of an extended strategy set, the initial population composition (seeding), the differences in pay-offs and finally different parameter spaces. I investigate the effects of these factors on the evolvability of both honest and mixed cheating strategies. I show that both honest and cheating equilibria readily evolve and that the investigated parameter range and the seeding of the starting populations have the greatest influence on the outcome.

**Conclusions:**

Both honest signalling and polymorphic cheating equilibria are more likely to evolve from a narrow strategy set than from a random mixture of strategies. A large potential strategy set is not a setback for the evolution of communication -honest or cheating- as long as the initial population is seeded with only a few strategies. In addition, different sections of the parameter space show consistently different behaviour. Thus, frequency dependent selection has the potential to explain various empirical observations that show consistent differences in aggressive behaviour.

**Electronic supplementary material:**

The online version of this article (10.1186/s12862-017-1112-y) contains supplementary material, which is available to authorized users.

## Background

Honest signalling has dominated discussions of animal signalling systems in the last few decades [1, 2]. However, it has been clear since early studies that animal communication need not be honest all the time [3, 4]. There is a growing body of literature on the use of dishonest signals in nature, in organism from microbes [5] to humans [6, 7]. Such deceptive signals can be used within or between species (intra vs. interspecific cheating). Between species these deceptive signals include various forms of mimicry (see review [8]): Batesian and Müllerian mimicry in butterflies [9, 10] and in fish [11]; myrmecomorphy in insects [12]; aggressive mimicry [13, 14]; and mimicry in cleaner-client systems [15]. Within species these deceptive signals include bluffing in the context of aggressive communication in stomatopods [16] fiddler crabs (*Uca annulipes*) [17], and American goldfinches (*Carduelis tristis*) [18]; and sexual mimicry in many species such as bluegill sunfish (*Lepomis macrohirus*) [19, 20], damselflies (*Ischnura ramburi*) [21] and red-sided garter snakes (*Thamnophis sirtalis parietalis*) [22]. A growing number of theoretical papers attempt to explain the presence of cheating in signalling systems both in general and in the context of aggressive communication [23–29].

Explaining honest communication or even a mixture of honest and dishonest signals presents a special challenge in the context of aggressive communication [30, 31] because a clear-cut conflict of interest exists between participants. Early theoretical models [32] have been highly sceptical about the possibility of honest signalling during aggressive interactions. Enquist [33] was the first to show that conventional signals can be honest and evolutionarily stable in this context. Enquist’s model [33] can be seen as a modified version of the Hawk-Dove game [32] with a free communication round in which players can be weak or strong. Players know their own states, but their states cannot be seen by observers. Enquist asked whether cost free signals emitted at the communication stage can reliably transmit information about this unobservable quality. To answer this question, he investigated two global strategies (see detailed definitions below): to be honest and to lie (cheat). He proposed an honest equilibrium where weak and strong individuals use different signals, and weak individuals avoid fighting with strong individuals. Cheaters are weak individuals who mimic the signal of the strong individuals. Enquist has shown that as long as potential cheaters are unable to flee from the attack of honest strong individuals, there is a fighting cost threshold that prevents the spread of cheating in an honest population. Both the model and the results were ground-breaking at that time, because Enquist was the first to show that variation in behaviour can be a reliable signal and also that the receiver’s response can maintain the signal’s honesty.

Enquist’s model, like most signalling games, assumes consistent individual variation between individuals. Animal personality research is a newly emerging and rapidly growing field with the primary goal of studying such individual differences ([34, 35]). Consistent individual variation has been shown to exist in many contexts, including aggression ([36, 37]). Individual variation also appears to carry over to signalling, and some examples of this phenomenon can be classified as cheating. For example, in recent studies Akcay and colleagues [38, 39] have found consistent “under” and “over” signalling behaviour in which aggressive individuals underuse, and less aggressive individuals over-use, threat displays. Potential examples of cheating are also found in other species, such as fiddler crabs [17] and goldfinches [18]. Despite extensive animal personality research, theoretical models attempting to explain the source of variation are rare. [40]. As a result, current empirical findings are still challenging to explain. In this paper, I suggest that frequency dependent-selection can offer an explanation for many of these observations.

Számadó [24] has proposed frequency dependent selection to explain cheating behaviour in Enquist’s game; he has shown that honest and cheating strategies can co-exist in a mixed equilibrium. This mixed equilibrium can be implemented in two different ways: (i) either a fixed proportion of weak individuals cheat (*p*) and the remaining portion remains honest (1-*p*), (ii) or each weak individual cheats with a probability *p* and stays honest with probability 1-*p*. Later Szalai & Számadó [25] (abbreviated as SS09) utilised an extended version of this game with individual based simulations to investigate other possible strategies, such as strong cheaters or individuals who always attack or always flee while ignoring signals (see Additional file 1: Appendix 1 for detailed definitions of these strategies). They have found a diverse set of honest and cheating equilibria that have different combinations of these strategies. However, the validity of this type of equilibrium has been challenged by a later study. First, Hamblin & Hurd [41] concluded that no honest equilibrium evolved; in fact, no signalling equilibrium -mixed or pure- evolved in the first place when using random or close to random initial populations. Later, Helgesen et al. [42] (abbreviated as H13) heavily criticised Számadó [24] and Szalai & Számadó [25] for using a limited strategy set (i.e., there are more than eight possible strategies in Enquist’s game) and slightly different pay-offs relative to those in previous versions [43]. They reiterated the conclusion of Hamblin & Hurd [41] and also claimed that no equilibrium with mixed cheating evolves in individual based simulations. They went as far as to claim, “Intuition and common sense have it that animals communicate using ambiguous threat displays that have an underlying probabilistic mixed strategy type of mechanism, but there remains no working game theoretical model of such a communication system” (Helgesen et al., [42] abstract).

It is important to resolve this controversy to decide whether frequency dependent selection can serve as a valid explanation for individual differences in aggressive contexts. Unfortunately, Helgesen et al. [42] used a completely different set of modelling assumptions than Szalai & Számadó [25], thus making it impossible to determine whether the effect claimed by Helgesen et al. [42] (i.e. lack of communication and mixed cheating) is the result of the introduction of a new, larger strategy set and a modified pay-off matrix or the result of other modelling assumptions. Here, I re-investigate those claims by using the strategy set and the pay-off matrix suggested by Helgesen et al. [42] and I also investigate the role of initial population composition and parameter space; otherwise, I use the modelling setup and assumptions of Szalai & Számadó [25].

## Methods

**Fig. 1:**
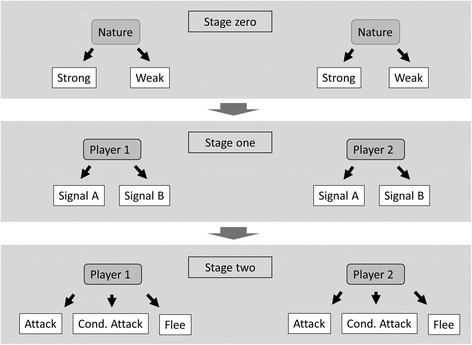
Schematic description of the Enquist game [33]. Stage zero: Nature picks a state for the contestants; this stage is hidden from other players. Stage one: each contestant picks a signal, A or B. Stage two: each contestant picks a behaviour as a response to the signal: Flee (F), Conditional Attack (CA) or Attack (At)

Enquist’s model [33] is a symmetric game of aggressive communication where two animals fight for the same resource. The players can be weak or strong, where *q* and 1-*q* give the frequencies of weak and strong individuals respectively. Each player knows its own state; however, the state of the opponent remains hidden. The game can be divided into three stages (see Fig. 1): (i) Nature decides the state of each player, (ii) each player can choose between two signals A or B, these signals are assumed to be free of production cost; finally (iii) each player can choose between three actions: flee, attack or attack conditionally. Conditional attack implies that the player waits for the opponent to withdraw and it attacks only if the other player stays to fight. Let *V* denote the value of the contested resource, this resource cannot be divided between the contestants. There are four cost parameters associated with fighting behaviour in the model. Enquist [33] assumed that a strong individual can always beat a weak individual, where *C*_*SW*_, and *C*_*WS*_ denote the cost of fighting for strong and weak player respectively. It is further assumed that the cost suffered by the weak player is larger than the cost paid by the strong one, hence the following relation holds: *C*_*WS*_ > *C*_*SW*_. Weak or strong individuals fighting between each other has an equal chance to win a fight, where *C*_*WW*_ and *C*_*SS*_ denote the expected costs. Overall, we assume the following relations between these costs: *C*_*WS*_ > *C*_*WW*_, *C*_*SS*_ > *C*_*SW*_. There are three cost parameters associated with fleeing in the model. Let *F*_*f*_ denote the cost of fleeing and *F*_*A*_, denote the cost of attacking a fleeing opponent. Finally, let *F*_*P*_ denote the cost of waiting when the opponent attacks unconditionally. It is usually assumed that these costs are small compared to fighting costs, i.e.: *C*_*sw*_ > *F*_*A*_, *F*_*P*_ [43].

Two more important considerations are involved: the source of variation and the strategy set available to the players. Enquist’s [33] model makes sense only if there is a polymorphism of weak and strong individuals. Enquist’s model [33] assumes a fixed 0.5 ratio between weak and strong individuals, whereas both Hurd’s model (1997) and later Számadó’s [24] first model consider a fixed ratio between 0 and 1. Számadó’s [24] second model further relaxes of this assumption by having a ratio that can change during the course of evolution. Szalai & Számadó’s model [25] followed Számadó’s [24] second model, in which the frequency of the alleles that regulate the ratio of strong to weak individuals is an evolutionary variable. In contrast Helgesen et al. [42] have used a fixed ratio of weak vs. strong individuals that is randomised for every play. Thus, whereas the models of Enquist [33], Hurd [43], and Helgesen et al. [42], and Számadó [24] first model, assume an exogenous explanation for the polymorphism of weak and strong individuals, Számadó’s [24] second model and Szalai & Számadó’s model [25] provide an endogenous explanation. Notably, the model implemented by Szalai & Számadó [25] is not a choice-of-state model, as has been erroneously claimed by H13: the chance of playing ‘strong’ or ‘weak’ is regulated by the alleles of a gene; thus, it is not up to individual choice. Because Szalai and Számadó [25] investigated a model with an endogenous explanation, here, I also implement the same version.

The original model consists of only two global strategies (honesty vs. cheating; Enquist, [33]. Enquist investigated the following honest global strategy denoted S (Enquist, [33]; p. 1155):

“*If strong, show A; if the opponent also shows A attack and if the opponent shows B, repeat A and attack only if it does not withdraw immediately. If weak show B and give up if the opponent shows A and attack if the opponent shows B*.”

The evolutionary stability of this honest global strategy *S* was investigated against a simple cheating type in which weak individuals show A instead of B. This cheating strategy was not explicitly defined by Enquist; the corresponding global strategy can be written up as follows (Számadó [24]; p. 222):

“*Display always A in the first round, regardless of strength; then in the second round if strong attack unconditionally if opponent shows A or wait until opponent flees if it has shown B; if weak withdraw if opponent signals A or wait until opponent flees if it has shown B.*”

**Fig. 2:**
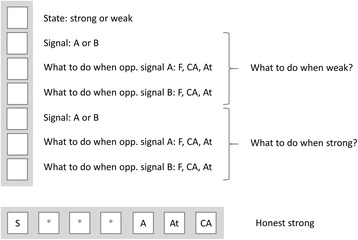
Schematic representation of the coding of the behaviour of individuals. Each individual has seven genes: the first gene represents the state of the individual (weak or strong), the next three and the last three encodes the behaviour of the individual depending on whether the state of the individual is weak or strong respectively. Out of these three genes the first gene gives which signal to use; the second gene encodes which behaviour to use as a response to signal A; and finally, the last gene encodes which behaviour to use as a response to signal B. W: weak; S: strong; F: Flee; CA: Conditional Attack; At: Attack. Asterisks denote silent regions which do not influence the behaviour of the given individual. The “genom” of an individual playing the Honest Strong strategy is at the bottom as an example

Figure 2 shows a schematic representation of the potential strategies and gives Honest Strong as an example. Each individual has 7 genes. The first one encodes the strength of the individual (weak or strong). The next three encode behaviour when weak: (i) signal when weak (A or B), (ii) response to signal A when weak (flee, conditional attack, attack), (iii) response to signal B when weak (flee, conditional attack, attack); finally, the last three genes encode behaviour when strong: (v) signal when strong (A or B), (vi) response to signal A when strong (flee, conditional attack, attack), and finally (vii) response to signal B when strong (flee, conditional attack, attack).

**Fig. 3:**
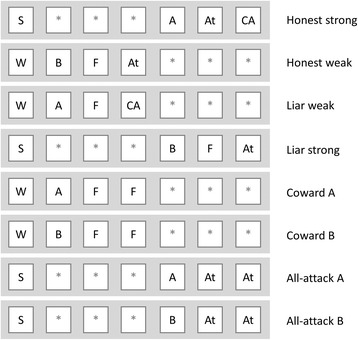
Schematic representation of the eight behavioural strategies that were used in the Szalai and Számadó model [25]. W: weak; S: strong; F: Flee; CA: Conditional Attack; At: Attack. Asterisks denote silent regions which do not influence the behaviour of the given individual

Szalai and Számadó investigated eight strategies, see Fig. 3 for representations and Additional file 1: Appendix 1 for definitions of these strategies. There are, however, more than eight strategies in Enquist’s game, and the full set has been investigated by Helgesen et al. [42] (see Additional file 1: Appendix 2). It is important to note that altough on paper there are 324 (18 × 18) possible pure strategies in the model, most of these strategies are redundant if the actual behaviour of any individual is examined. Because individuals are weak or strong for life, in the current implementation of the model, therefore half of their genes will be never expressed (see Figs. 2 and 3). When *classifying* the behaviour of the individuals, these inactive genes can be safely ignored, thus greatly simplifying the analysis. Notably, these inactive alleles are still present (even if they are not used for classification), and they can be turned on by mutation. Accordingly, I will consider only 36 strategies in the further analysis (see Additional file 1: Appendix 2).

Szalai and Számadó [25] investigated only 8 strategies; however, they investigated more than 10,000 parameter combinations (see Table 1). Hamblin & Hurd [41] investigated all of the possible 324 strategy combinations; however, they investigated only a small fraction of the possible parameter space (12 parameter combinations, see Table 2). Additionaly, the two groups -Szalai and Számadó [25] and Helgesen et al. [42]- have used slightly different versions of the pay-off matrix; this difference is most noticeable at the flee vs. flee option (for comparison of pay-offs see Table 3). Here, I investigate both versions. Here I also change the genetic representation of the strategy set from Szalai & Számadó [25] to the one suggested by Helgesen et al., [42] (see further details in Fig. 2 and Additional file 1: Appendix 2) to allow the full strategy set to evolve.

**Table 1:** Szalai and Számadó [25] parameter space

Parameter	Start	End	Step
*V*	1	34	3
*C*_*SS*_	10	35	5
*C*_*WW*_	2	32	5
*C*_*SW*_	10	35	5
*C*_*WS*_	2	32	5
*F*_*p*_	0	min(*C*_*SS*_, *C*_*WW*_, *C*_*SW*_, *C*_*WS*_)	3

Where *V* denotes the value of the contested resource, *C*_*WW*_ and *C*_*SS*_ denote the expected costs of a fight between two weak and two strong individuals respectively; *C*_*SW*_, and *C*_*WS*_ is the expected cost for a strong animal fighting a weak and vice versa; and finally, *F*_*p*_ denotes the cost of waiting if the opponent attacks unconditionally. The following relation holds between these costs: *C*_*SS*_ > *C*_*WS*_ > *C*_*WW*_, and *C*_*SS*_ > *C*_*SW*_ > *C*_*WW*_; combinations that do not fit these conditions were not investigated. All in all, cc 10,000 parameter combinations were investigated

**Table 2:** Helgesen et al., [42] parameter space

Parameter	Standard E85 model	Choice-of-state model
*V*	100	100
*C*_*SS*_	15	100, 200
*C*_*WW*_	15	15
*C*_*SW*_	15	75
*C*_*WS*_	50, 70	50, 75
*F*_*a*_	5	5
*F*_*p*_	5	5
*F*_*f*_	0, 5	0, 5

Where *V* denotes the value of the contested resource, *C*_*WW*_ and *C*_*SS*_ denote the expected costs of a fight between two weak and two strong individuals respectively; *C*_*SW*_, and *C*_*WS*_ is the expected cost for a strong animal fighting a weak and vice versa; and finally, *F*_*p*_ denotes the cost of waiting if the opponent attacks unconditionally

**Table 3:** Combined payoffs matrix

		Opponent strength					
			Strong			Weak	
Ego Strength		Attack	Cond. attack	Flee	Attack	Cond. attack	Flee
	Attack	0.5 *V*–*C*_*SS*_	0.5 *V*–*C*_*SS*_	*V*-*F*_*A*_	*V*-*C*_*SW*_	*V*-*C*_*SW*_	*V*-*F*_*A*_
Strong	Cond. attack	0.5 *V*–*C*_*SS*_-*F*_*P*_	0.5 *V*–*C*_*SS*_	*V*	V-*C*_*SW*_-*F*_*P*_	*V*-*C*_*SW*_	*V*
	Flee (S00)	***- C***_***SS***_	0	**0.5** ***V*** **–** ***C***_***SS***_	**-** ***C***_***SW***_	0	0.5 *V*
	Flee (SS09)	***- C***_***SS***_ ***+ F***_***f***_	0	**0.5** ***V*** **–** ***C***_***SS***_	**-** ***C***_***SW***_ ***+ F***_***f***_	0	0.5 *V*
	Flee (H13)	***- C***_***SS***_	0	**0.5** ***V***	**-** ***C***_***SW***_	0	0.5 *V*
	Attack	-*C*_*WS*_	-*C*_*WS*_	*V*-*F*_*A*_	0.5 *V*–*C*_*WW*_	0.5 *V*–*C*_*WW*_	*V*-*F*_*A*_
	Cond. attack	-*C*_*WS*_-*F*_*P*_	-*C*_*WS*_	*V*	0.5 *V*–*C*_*WW*_-*F*_*P*_	0.5 *V*–*C*_*WW*_	*V*
Weak	Flee (S00)	**-** ***C***_***WS***_	0	0.5 *V*	**-** ***C***_***WW***_	0	**0.5** ***V*** **–** ***C***_***WW***_
	Flee (SS09)	**-** ***C***_***WS***_ **+** ***F***_***f***_	0	0.5 *V*	**-** ***C***_***WW***_ ***+ F***_***f***_	0	**0.5** ***V*** **–** ***C***_***WW***_
	Flee (H13)	**-** ***C***_***WS***_	0	0.5 *V*	**-** ***C***_***WW***_	0	**0.5** ***V***

Bold letters denote differences in pay-offs in the Flee choice: Flee(S00) Számadó [24], Flee(SS09) Szalai & Számadó [25], Flee(H13) Helgesen et al., [42]. *V*: value of the contested resource; *C*_*SS*_, *C*_*WW*_: expected cost of fight between equal opponents; *C*_*SW*_: cost for strong individual to beat weak one; *C*_*WS*_: cost to weak individual when beaten by strong one; *F*_*f*_: cost of fleeing; *F*_*A*_: cost of attacking fleeing opponent; *F*_*P*_: cost of waiting if the opponent attacks unconditionally

The feasibility of the evolvability of honest and cheating equilibria is assessed by individual based simulations. Here I investigate the effect of differences in (i) pay-offs and (ii) the effect of initial composition on the evolutionary trajectories of these populations, using the extended strategy set as suggested by Helgesen et al. [42] while keeping the other modelling assumptions the same as those in Szalai and Számadó [25] (i.e., number of fights and source of variation). To investigate the effect of initial strategy distribution, I use two different setups: either (i) seeding the population randomly from all the possible 36 strategies, or (ii) using the eight strategies used by SS09 to seed the initial population in order to compare the effects of switching from 8 strategies to the full strategy space.

All in all, I investigate evolvability with the following four different setups: (i) the initial population consists of random strategies drawn from the full set using the SS09 pay-offs; (ii) the initial population consists of random strategies drawn from the full set using the H13 pay-offs; (iii) the initial population consists of eight strategies used by SS09 using the SS09 pay-offs; and finally, (iv) the initial population consists of eight strategies used by SS09 using the H13 pay-offs. I use the full strategy set in all of these investigations as suggested by H13 (i.e. any of the possible strategies can evolve even if they are not present in the initial distribution), and I investigate parameter regions from the SS09 and H13 studies.

Of the vast parameter space investigated by SS09 I investigate only those sections where main signalling equilibria evolved in the original study (see Additional file 2: Dataset 1). Szalai and Számadó have [25] found six such equilibria: (i) Honest-strong, Honest-weak, which is the traditional honest signalling outcome (SS09 code: 3; current code: <30,2>); (ii) Honest-strong, Liar-strong, this is an “all-strong” honest signalling outcome where strong individuals signal differences in intentions (fight vs. flee) with the use of the signal (SS09 code: 5; current code: <30,20>); (iii) Honest-strong, Honest-weak, Liar-weak, which is the “traditional” cheating scenario (SS09 code: 11; current code: <30,2,14>); (iv) Honest-strong, Liar-strong, Liar-weak, which can be viewed as an “all-strong” cheating scenario in which the weak strategy imitates one of the strong ones (SS09 code: 13; current code: <30,20,14>); (v) Honest-strong, Honest-weak, Liar-strong, Liar-weak, which is a “full-scale” cheating scenario (SS09 code: 15; current code: <30,2,20,14>);.and finally, (vi) Honest-strong, Honest-weak, Liar-weak, Coward, this is an “all-strong” cheating scenario with cowards (SS09 code: 27; current code: <30,2,14,8,17>). The “current code” gives the code of pure strategies (according to Additional file 1: Appendix 2) supporting the given polymorphic equilibrium. These parameter regions are denoted by the code of the strategy combination used by SS09 (code3, code5, etc.). Out of these parameter regions 500–500 parameter combinations were drawn randomly and 10 independent runs were made with each combination. All in all, 3000 parameter combinations were investigated from the SS09 study. See Additional file 2: Dataset 1 for the parameter combinations and results of the SS09 study; and Additional file 3: Dataset 2 for the details of the 6 parameter regions described above. Finally, I investigate the evolvability of mixed cheating with the H13 parameter range as well (see Table 2), using the same modelling assumptions and same variation in pay-offs and initial strategy distributions as for the SS09 parameter space. See Table 4 for a comparison of the main differences between the two studies and for the general setup of the current study. Further details of the computer simulations are described in Additional file 1: Appendix 3, and Additional file 4: Table S1 summarises all of the investigated scenarios.

**Table 4:** The main differences between the Szalai and Számadó [25] (SS09) and the Helgesen et al. [42] (H13) studies

	SS09	H13	current study
number of strategies	8	18 × 18	36
pay-offs	Table 3. Flee(SS09)	Table 3. Flee(H13)	both
parameter range	cc. 10,000	12	both
initial population	8 strategies	18 × 18 strategies	8 or 36
variation in state	endogenous	fixed ratio	endogenous

## Results

**Fig. 4:**
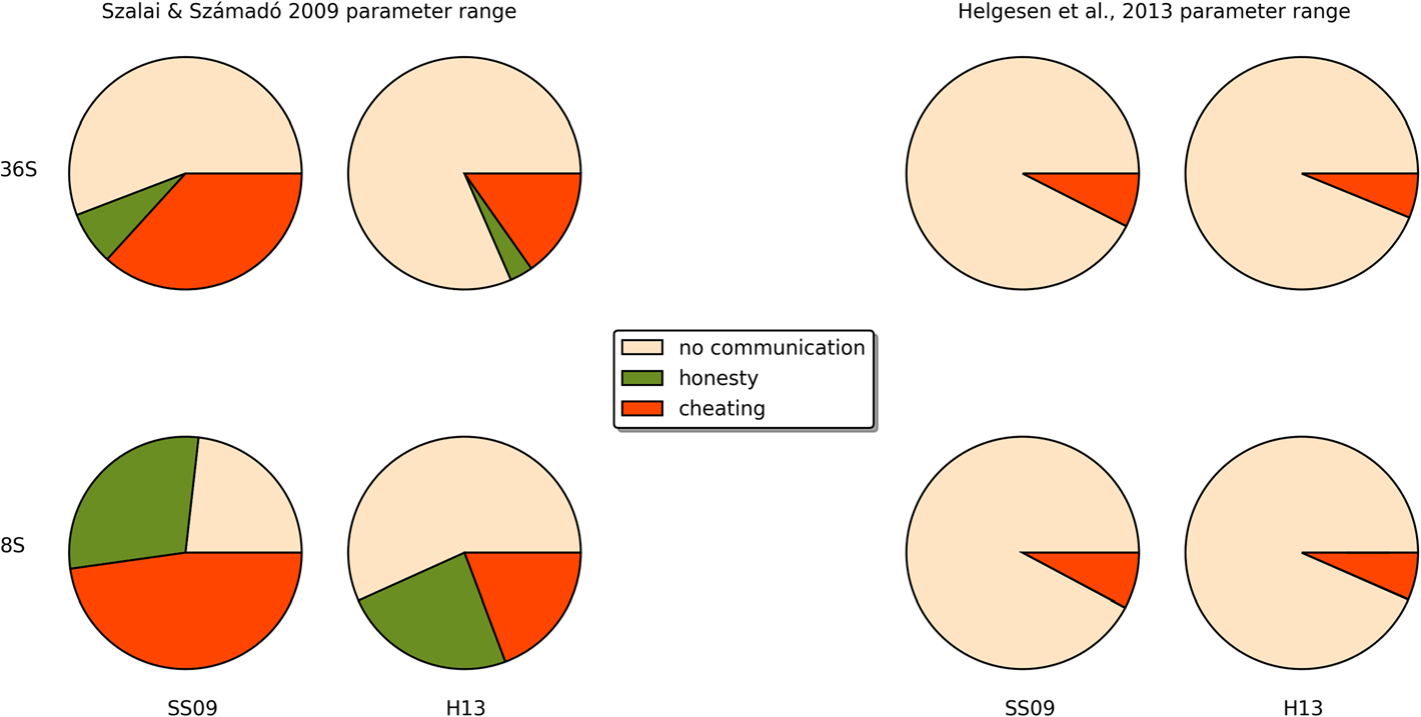
Different types of outcomes observed in the individual simulations: (i) no-signalling: white; (ii) honest signalling: green; (iii) cheating equilibria: orange. 36S and 8S denotes seeding with random initial strategy distribution and seeding with the eight strategies used by Szalai & Számadó [25] respectively; finally, SS09 denotes the pay-offs used by Szalai & Számadó [25]; while H13 denotes the pay-offs used by Helgesen et al. [42]

**Fig. 5:**
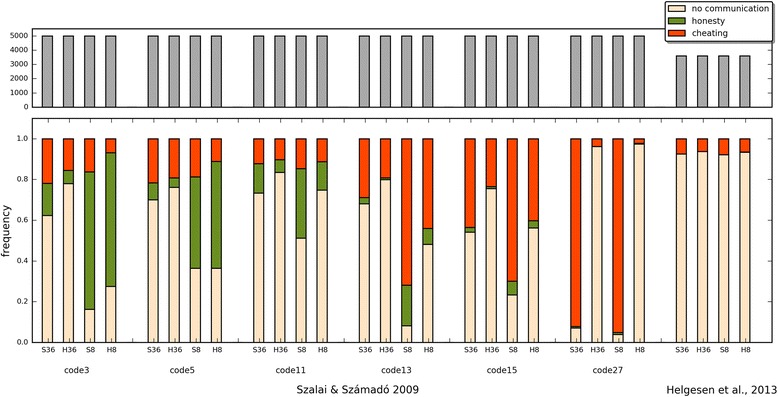
The distribution of equilibrium strategies as a function parameter range, pay-offs and initial strategy composition. Colour codes are as follows: (i) no-signalling: white; (ii) honest signalling: green; (iii) cheating equilibria: orange. Thirty-six and 8 denotes random initial strategy distribution and populations seeded with the eight strategies used by Szalai & Számadó [25] respectively; S: denotes the pay-offs used by Szalai & Számadó [25]; while H denotes the pay-offs used by Helgesen et al., [42]. Parameter ranges are Szalai & Számadó, [25] and Helgesen et al., [42]; code* denote a list of parameter combinations where the following strategies evolved in Szalai & Számadó [25]: code3: Honest-strong, Honest-weak; code5: Honest-strong, Liar-strong; code11: Honest-strong, Honest-weak, Liar-weak; code13: Honest-strong, Liar-strong, Liar-weak; code15: Honest-strong, Honest-weak, Liar-strong, Liar-weak; code27: Honest-strong, Honest-weak, Liar-weak, Coward. The upper panel shows the total number of runs made in the given parameter range

The evolutionary trajectories of the populations are clearly strongly influenced by all investigated factors. The frequencies of honest, cheating and no-signalling equilibria are given in Additional file 4: Table S2 (the full dataset is available at Mendely data as Számadó2017_results.csv). Figure 4 shows the results of the computer simulations as a function of (i) pay-off (SS09 vs. H13), (ii) initial population composition (random vs. eight strategies) and (iii) parameter ranges (SS09 vs. H13). Figure 5 further classifies the results as a function of the parameter sets of the SS09 study, i.e. code3, code5, code11, code13, code15, code 27.

### Pay-offs

It is clear that the SS09 pay-offs are indeed more favourable for polymorphic cheating equilibria than the pay-offs proposed by H13. However, changing the pay-offs does not explain the completely opposite outcome, as such polymorphic cheating equilibria still evolve with the H13 pay-offs.

### Initial population composition

**Fig. 6:**
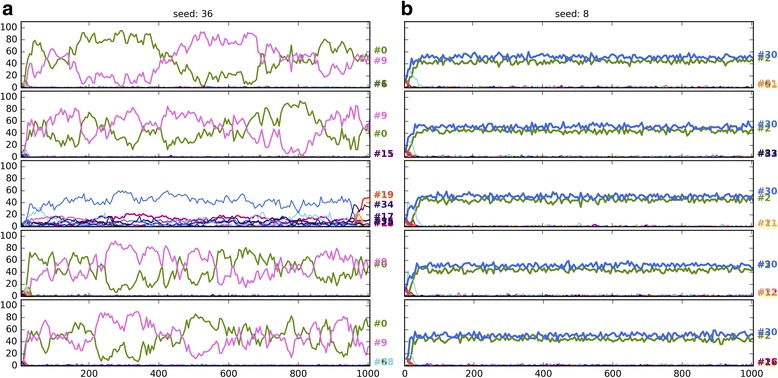
Ten independent timelines of the parameter combination: 984 (parameter combinations are defined in SI file 2). Pay-offs: H13; parameter region: code3. Each figure shows five-five independent runs with the same parameter combination: (**a**, left column) 36 strategy seed, (**b**, right column) 8 strategy seed. Strategy codes are displayed on the right. *V* = 13.0, *Css* = 20.0, *Cww* = 7.0, *Cws* = 15.0, *Csw* = 17.0, *Ff* = 0.0

**Fig. 7:**
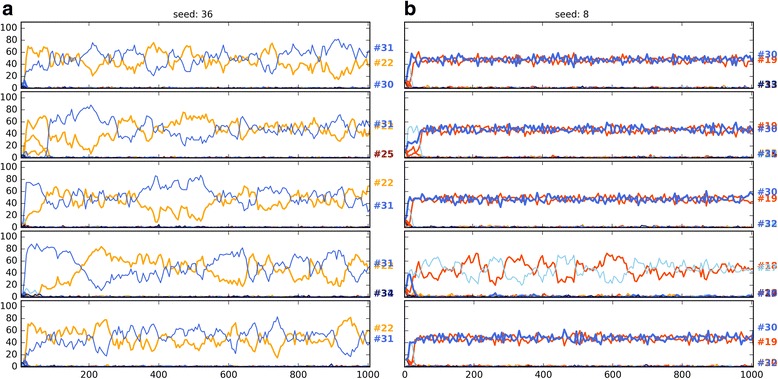
Ten independent timelines of the parameter combination: 6269. Pay-offs: H13; parameter region: code5; (**a**) 36 strategy seed, (**b**) 8 strategy seed. V = 34.0, Css = 30.0, Cww = 12.0, Cws = 30.0, Csw = 12.0, Ff = 0.0

**Fig. 8:**
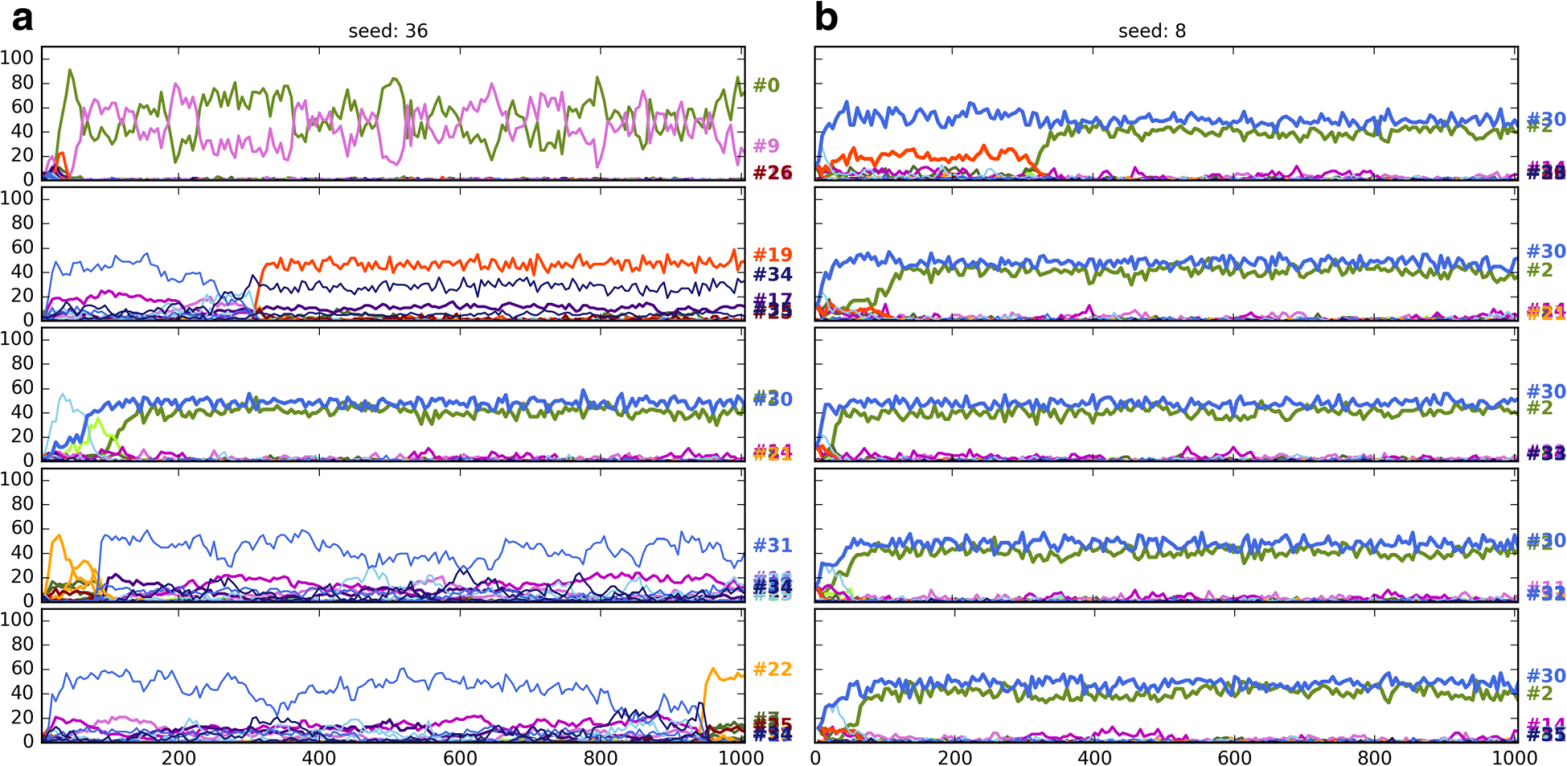
Ten independent timelines of the parameter combination: 4226. Pay-offs: H13; parameter region: code11; (**a**) 36 strategy seed, (**b**) 8 strategy seed. *V* = 22.0, *Css* = 35.0, *Cww* = 12.0, *Cws* = 30.0, *Csw* = 27.0, *Ff* = 9.0

Seeding the populations with only 8 strategies (8S) instead of 36 (36S) favours the evolution of honest or dishonest polymorphic equilibria depending on the parameter regions (Fig. 5). Notably, with random seeding, the frequency of honest strategies takes a hit as a strong or even stronger than that of mixed cheating. In other words, random seeding strongly favours the evolution of no-signalling regardless of the pay-offs. Figures 6, 7 and 8 show examples of when change of seeding results in no-signalling outcomes with the same pay-offs and parameter combinations in three regions (code3, code5, code11).

### Parameter regions

**Fig. 9:**
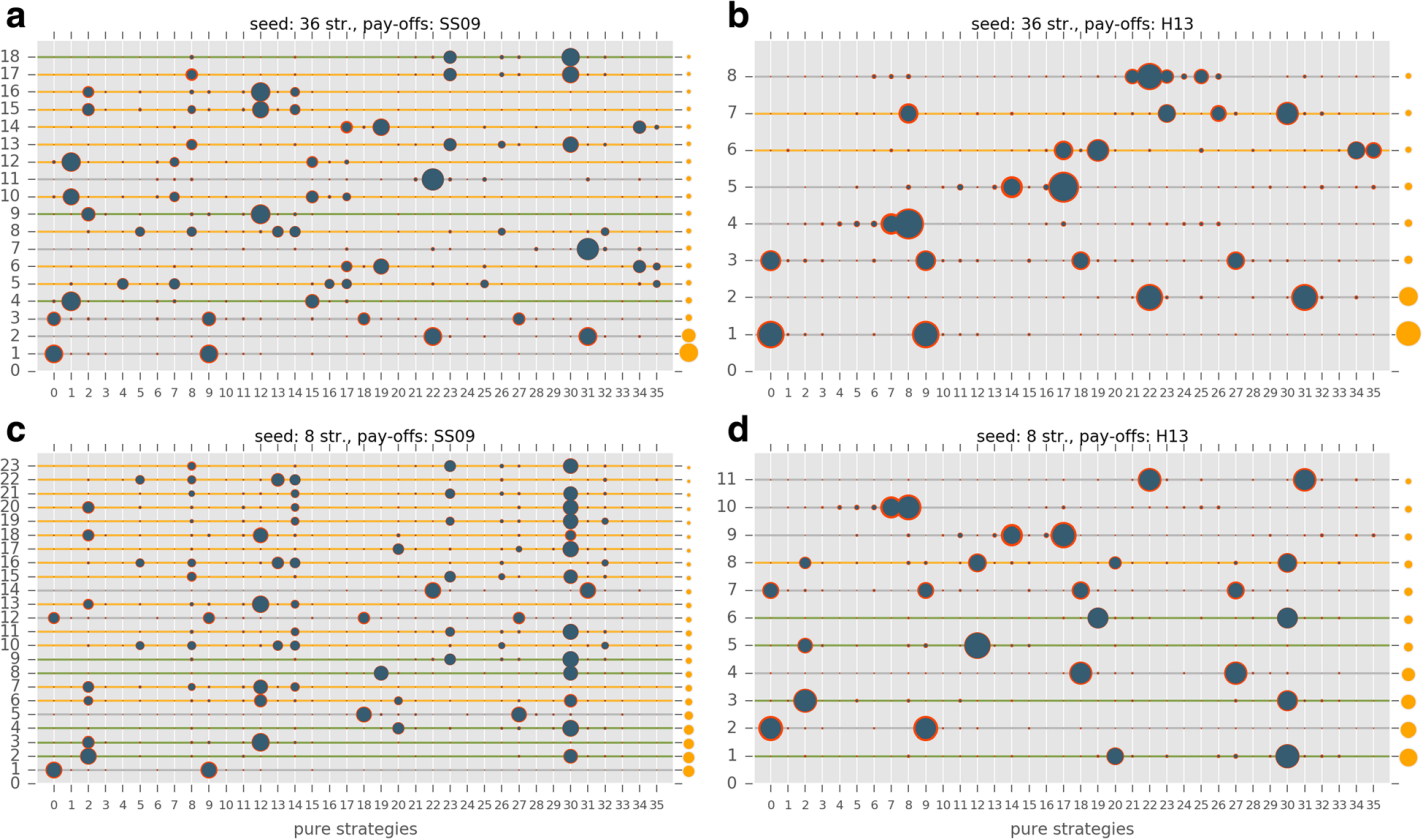
The composition and the frequency of the major equilibria (found in more than 0.01% out of 73,530 runs) that evolved using the SS09 parameter range in four different scenarios: **a** SS09 pay-offs, random initial strategy composition; **b** H13 pay-offs, random initial strategy composition; **c** SS09 pay-offs, seeded with 8S of SS09; **d** H13 pay-offs, seeded with 8S of SS09. Orange dots on the right side show the observed frequencies of these strategy combinations, values can be found in Additional file 4: Table S3. The size of the blue dots is proportional to the average frequency of the given pure strategy at the equilibrium, the red circle is proportional to the standard deviation. Green, yellow and grey lines denote honest, cheating and no-signalling equilibria respectively

Populations respond differently to changes in pay-offs in different parameter regions. On the one hand, the outcome is not sensitive to the changes in pay-offs (SS09 vs. H13) in those regions where honest strategies dominate with 8S seeding (Fig. 5. code3, code5). On the other hand, the outcome is sensitive to the changes in pay-offs in parameter regions where dishonest polymorphic equilibria dominate with 8S seeding (Fig. 5. code11, code13, code15, code27). A prime example of this phenomenon is region code27 where changes in pay-offs reverse the results, from almost complete domination of polymorphic equilibria to almost complete absence of these types of equilibria. Regarding the parameter range, the worst-case scenario for mixed equilibria is the H13 parameter region used by Helgesen et al. [42]; in this region (Fig. 5. Helgesen et al.), polymorphic equilibria are almost absent even with the beneficial pay-offs (SS09) and favourable seeding (8S). Additional file 5: Figure S1 and Additional file 6: Figure S2 provide examples of individual timelines from the SS09 and H13 parameter regions respectively. Figure 9 shows the frequency and the composition of the major equilibria (found in more than 0.01% of the runs) that evolve with the SS09 parameter set (see Additional file 4: Table S3). The combination of the H13 pay-offs and random initial population is clearly the most unfavourable combination for the evolution of cheating (see Fig. 9b). Notably, this combination is also the least favourable for the evolution of honest equilibria. Switching to the SS09 pay-offs yields honest and cheating equilibria (Fig. 9a) but the most frequently observed outcomes are still populations with no-signalling. Switching to initial populations seeded with the eight strategies used by SS09 results in a drastic change: honest equilibria are amongst the most frequent outcomes regardless of the pay-offs (<20,30 > and <2,30>; see Fig. 9c and d). Cheating also evolves more readily and it is favoured more by the SS09 pay-offs (see Fig. 9a vs. b and c vs. d).

**Fig. 10:**
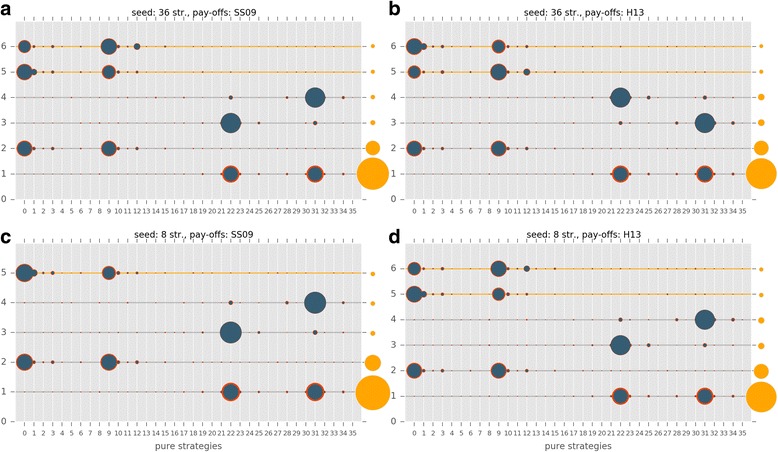
The composition and the frequency of the major equilibria (found in more than 0.01% out of 3600 runs) that evolved in the H13 parameter range in four different scenarios: **a** SS09 pay-offs, random initial strategy composition; **b** H13 pay-offs, random initial strategy composition; **c** SS09 pay-offs, seeded with 8S of SS09; **b** H13 pay-offs, seeded with 8S of SS09. Orange dots on the right side show the observed frequencies of these strategy combinations, values can be found in Additional file 4: Table S4. The size of the blue dots is proportional to the average frequency of the given pure strategy at the equilibrium, the red circle is proportional to the standard deviation. Green, yellow and grey lines denote honest, cheating and no-signalling equilibria respectively

Figure 10 shows the frequency and the composition of the major equilibria that evolved with the H13 parameter set (see Additional file 4: Table S4). Again, it is clear that the H13 set is the most unfavourable parameter set for both mixed cheating and honest signalling; It is also worth to note that the Helgesen et al. [42] parameter region is the only region which is completely insensible to changes in the modelling assumptions; it is the only parameter region where no-signalling dominates regardless of the seeding or the pay-offs (Figs. 4, 5 and 10).

Over all, the most favourable combination of pay-offs and seeding for honest and cheating equilibria to evolve is provided by the original SS09 setup (i.e. the combination of the SS09 pay-offs and 8S seeding). Dishonest polymorphic equilibria still evolve in most of the SS09 parameter regions with the H13 pay-offs. In contrast, the least favourable combination of pay-offs, seeding and parameter range is the combination used in the Helgesen et al. [42] study: H13 pay-offs with random seeding of 36 strategies, using the H13 parameter range.

## Discussion

Here, I investigated the effects of four modelling assumptions on the evolutionary trajectories of populations playing a simple game of aggressive communication. The factors investigated were: the overall strategy set, the initial strategy composition, the pay-off matrix and finally the parameter space. All of these factors influence the outcome, yet their importance varies. The least important factor is perhaps the introduction of the *full strategy set*. Both honest and cheating strategies readily evolve if all the modelling assumptions of SS09 hold constant otherwise (and in fact they are amongst the most frequent strategies, see Fig. 9c).

The next most important factor is the *pay-offs*. Whereas the SS09 pay-off matrix indeed favours the evolution of polymorphic dishonest equilibria (i.e. cheating), switching to the H13 pay-offs never results in the complete disappearance of mixed cheating (see Figs. 4 and 5). Switching to the H13 pay-offs does result in the disappearance of honest and cheating strategies from the most frequent strategies when the seeding consists of all the possible strategies (see Figs. 9a vs. b), however switching to the H13 pay-offs has a much smaller effect when the original seeding consists of 8 strategies (Fig. 9c vs. d).

Changing the *initial strategy composition* from random to the 8 strategies used by SS09 always results significant changes in the outcome regardless of the parameter space or pay-offs (see Figs. 9 and 10a, vs. c, and b, vs. d, respectively). In all cases this change favours the evolution of signalling: both honest and cheating equilibria are more frequent.

Finally, the *choice of parameter space* influences the results significantly. For example, when the H13 parameter space is used neither honest nor cheating equilibria evolve to be amongst the most frequent equilibria with random initial populations regardless of the pay-offs (Fig. 10a vs. b), whereas different regions of the SS09 parameter space favour the evolution of honest equilibria (code3, code5) or mixed cheating (code11, code13, code15; see Fig. 5).

Additionally, although most of the criticism by Helgesen et al. [42] concerned the changes in pay-offs, this change alone never results the complete disappearance of cheating equilibria (see Figs. 5 and 9). Contrary to the claims of H13, at least three modelling assumptions must be changed relative to SS09 to obtain a drastic decrease in cheating (and honest) strategies: the overall strategy set, the composition of the initial populations and the pay-offs (see Fig. 9b). All four assumptions (strategy set, initial strategy composition, pay-offs, parameter range) must be changed to obtain an almost complete disappearance of signalling, both honest and dishonest (see Figs. 4, 5 and 10) as reported by Helgesen et al. [42]. The results also show that despite the claims of Helgesen et al. [42] the parameter range they used in their study is not representative of the Szalai and Számadó [25] parameter range. Finally, while the critique of Helgesen et al. [42] was questioned the existence of mixed cheating, the changes introduced by Helgesen et al. [42] equally effect the evolvability of honest equilibria as well.

The current results show, in accordance with the previous results of Szalai and Számadó [25], that the evolutionary attractors of cheating as well as honest equilibria are more restricted than those of the no-signalling equilibria. This result, although hardly surprising, does suggest that these kinds of equilibria are unlikely to evolve out of populations using a *random set of initial strategies*. However, no one expects signalling to evolve out of a random set of behaviours; in fact, there is good reason to assume that signalling (honest or cheating) is preceded by cues that are informative in some way. This is the equivalent of already existing correlations, i.e. the equivalent of seeding populations with a more restricted strategy set [25]. Szalai & Számadó [25] provide a long discussion about these potential cues in the context of aggressive communication i.e. “frozen” first steps of fighting techniques (see also [44, 45]).

Finally, I discuss an empirical example in detail: the “soft song”. Soft songs are low-amplitude calls observed in a number of species, mostly in aggressive context [46]. Empirical studies have shown that among the song types associated with an aggressive context, such as song type switching, song matching, and soft-song [47], only soft song predicts the probability of attack. Soft song is a reliable predictor of attack in song sparrows (*Melospiza melodia*) [31, 48, 49], swamp sparrows (*Melospiza georgiana*) [50] and black-throated blue warblers (*Dendroica caerulescens*) [51]. Soft song has number of features that are consistent with the Enquist model: (i) it has a negligible production cost (signals in the Enquist model are assumed to be cost-free); (ii) the honesty of soft song is maintained by “receiver retaliation” [31, 49, 52], i.e., by the receiver’s reaction to the signal, as is the case in the Enquist model. It has recently revealed that individual variation exists in some species [38]. There are “over” and “under” signallers, such that less aggressive individuals signal more frequently, whereas some aggressive individuals signal less frequently. In the terminology of the current model, these behaviours are the equivalent of the Liar-strong and Liar-weak strategies. If the honest strategies are assumed to be present in the population, this scenario is the Honest-strong, Honest-weak, Liar-strong, Liar-weak equilibrium (c15; current code: <30,2,20,14>). Szalai and Számadó [25] found this combination amongst the six most frequent outcomes. This outcome disappears with the introduction of the H13 pay-offs (see Additional file 4: Table S3), a slightly different version is still observed with a different kind of dishonest weak strategy <30,2,20,12>. While Akcay and colleagues [38] list a number of possible explanations for the existence of under- and over-signalling, frequency dependent selection is not among the possible explanations. The current model, together with the previous results [24, 25], offers a potential explanation, namely, that frequency dependent selection may explain the existence of polymorphic equilibria in aggressive communication in which several strategies can co-exist at equilibrium, including honest and various cheating strategies (i.e. “over” and “under” signallers).

## Conclusions

Previous models have shown that mixed cheating can be explained by frequency dependent selection, that these equilibria can be evolutionarily stable [24] and that these equilibria can evolve under appropriate conditions [25]. The current study supports this conclusion as it shows that frequency-dependent selection can maintain a diverse set of strategies at equilibrium; thus, it has a currently underappreciated role in explaining diversity in nature in the context of aggressive communication. Out of the four factors investigated in the current study, the initial strategy composition and the choice of parameter space appear to be the most influential and to be equally important in determining the evolutionary trajectories of the populations. The current study shows that when searching for the origins of honest or dishonest signalling one must look for more than a random mixture of behaviours.

## Additional files

Additional file 1: Appendix 1–3. (PDF 368 kb)

Additional file 2: Dataset 1. Szalai and Számadó [25] results. (CSV 1421 kb)

Additional file 3: Dataset 2. Szalai and Számadó [25] parameter regions. (CSV 292 kb)

Additional file 4: Tables S1–S4. (PDF 728 kb)

Additional file 5: Figure S1. Individual timelines SS09 parameter set. (PDF 1820 kb)

Additional file 6: Figure S2. Individual timelines H13 parameter set. (PDF 11297 kb)

## Abbreviations

H13: Helgesen et al. [42]
SS09: Szalai and Számadó [25]
36S: Seeding the populations with 36 strategies
8S: Seeding the populations with 8 strategies
*C*_*SS*_: The cost of fighting for strong individual against a strong one
*C*_*SW*_: The cost of fighting for strong individual against a weak one
*C*_*WS*_: The cost of fighting for a weak individual against a strong one
*C*_*WW*_: The cost of fighting for weak individual against a weak one
*F*_*A*_: The cost of attacking a fleeing opponent
*F*_*f*_: The cost of fleeing
*F*_*P*_: The cost of waiting when the opponent attacks unconditionally
*q*: Frequency of weak individuals
*V*: The value of the contested resource
