# Supplementary material for: When honesty and cheating pay off: the evolution of honest and dishonest equilibria in a conventional signalling game
Source: BMC Evol Biol. 2017 Dec 28;17:270. doi: 10.1186/s12862-017-1112-y (PMC5745956; doi:10.1186/s12862-017-1112-y)
Supplement: Supplementary file 1 — Appendix 1–3. (PDF 368 kb) [file 12862_2017_1112_MOESM1_ESM.pdf]

# Supplementary Information

# When honesty and cheating pay off: the evolution of honest and dishonest equilibria in a conventional signalling game

Szabolcs Számadó

MTA TK "Lendület" Research Center for Educational and Network Studies (RECENS)

Hungary, Budapest, Tóth Kálmán u. 4. H-1097

## Appendix 1. Comparison of strategy sets

Szalai and Számadó [25] defined the strategy set investigated in their model as follows:

- Honest–strong (Shs): *give signal A; if opponent gives signal A then attack. If it gives signal B then wait until it flees. If it does not flee then attack (that is, after signal B use conditional attack)*.
- Honest–weak (Shw): *give signal B; if opponent gives signal A then flee. If it gives signal B then attack.*
- Liar–strong (strong) (Sls): *give signal B; if opponent gives signal A then flee. If it gives signal B then attack.*
- Liar–weak (weak) (Slw): *give signal A; if opponent gives signal A then flee; if it gives signal B then use conditional attack.*
- Coward A (weak) (ScA): *give signal A; flee regardless of the opponent's signal.*
- Coward B (weak) (ScB): *give signal B; flee regardless of the opponent's signal.*
- All-attack A (strong) (SaaA): *give signal A; attack regardless of the opponent's signal.*
- All-attack B (strong) (SaaB): *give signal B; attack regardless of the opponent's signal.*

**Appendix 2. The strategy set of the model.**

The specification and codes of the strategies that were used in the simulation and their common names. The first column gives the code of the strategy. The second column encodes the strength of the individual: weak (0) or strong (1). The third column encodes the signal: A (1) or B (0). The fourth column gives the response to signal A: attack (0), conditional attack (1) or flee (2); finally, the fifth column specifies the response to signal B: attack (0), conditional attack (1) or flee (2). Only the active genes are listed here i.e., the ones that correspond to the current strength of the individual. There is a mirror list of inactive genes (not shown here).

| code | strength | signal | response to signal B | response to signal A | common name |
|---|---|---|---|---|---|
| 0. | 0 | 0 | 0 | 0 | Weak-all-attack(0) |
| 1. | 0 | 0 | 0 | 1 | |
| 2. | 0 | 0 | 0 | 2 | Honest-weak |
| 3. | 0 | 0 | 1 | 0 | |
| 4. | 0 | 0 | 1 | 1 | |
| 5. | 0 | 0 | 1 | 2 | |
| 6. | 0 | 0 | 2 | 0 | |
| 7. | 0 | 0 | 2 | 1 | |
| 8. | 0 | 0 | 2 | 2 | Coward(0) |
| 9. | 0 | 1 | 0 | 0 | Weak-all-attack(1) |
| 10. | 0 | 1 | 0 | 1 | |
| 11. | 0 | 1 | 0 | 2 | |
| 12. | 0 | 1 | 1 | 0 | Liar-weak-bold |
| 13. | 0 | 1 | 1 | 1 | |
| 14. | 0 | 1 | 1 | 2 | Liar-weak |
| 15. | 0 | 1 | 2 | 0 | |
| 16. | 0 | 1 | 2 | 1 | |
| 17. | 0 | 1 | 2 | 2 | Coward(1) |
| 18. | 1 | 0 | 0 | 0 | All-attack(0) |
| 19. | 1 | 0 | 0 | 1 | |
| 20. | 1 | 0 | 0 | 2 | Liar-strong |
| 21. | 1 | 0 | 1 | 0 | |
| 22. | 1 | 0 | 1 | 1 | |

| | | | | | |
|---|---|---|---|---|---|
| 23. | 1 | 0 | 1 | 2 | |
| 24. | 1 | 0 | 2 | 0 | |
| 25. | 1 | 0 | 2 | 1 | |
| 26. | 1 | 0 | 2 | 2 | |
| 27. | 1 | 1 | 0 | 0 | All-attack(1) |
| 28. | 1 | 1 | 0 | 1 | |
| 29. | 1 | 1 | 0 | 2 | |
| 30. | 1 | 1 | 1 | 0 | Honest-strong |
| 31. | 1 | 1 | 1 | 1 | |
| 32. | 1 | 1 | 1 | 2 | |
| 33. | 1 | 1 | 2 | 0 | |
| 34. | 1 | 1 | 2 | 1 | |
| 35. | 1 | 1 | 2 | 2 | |

**Appendix 3. Computer simulations.**

The computer simulations are based on the methodology of Szalai and Számadó [25]. Ten independent runs were made with every parameter combination in the SS09 parameter regions, whereas 100 independent runs were made with every parameter combination in the H13 parameter region. Each run consists of 1000 iterations. During each iteration individuals fight and then reproduce. N=100 individuals are randomly selected to fight and they fight a $k$ number of randomly selected opponents where $k$ is a random number between 0 and 50. The fitness of an individual is based on its success competing for the resource ($V$). The fitness gain of a fight is calculated from the payoff matrix (Table 3). After the fight cycle reproduction takes place.

Reproduction is the same as in Szalai and Számadó (2009, Appendix). Individual strategies are coded by their genes. The population is divided into three parts based on a rank order of their fitness values, which they acquired during the fighting stage. These parts are as follows: first $N(1- P)$, the middle $N(2P - 1)$ and the last $N(1 - P)$ individuals. Out of these individuals the first $N(1- P)$ individuals produce two offsprings, while the next $N(2P - 1)$ individuals produce only one new individual. The remaining $N(1 - P)$ individuals die without leaving their genes to the next generation. Finally, there is an $m$ mutation probability with which genes can flip from one state to the other. This probability $m$=0.001 was fixed during the simulations for all genes.
