# Supplementary material for: When honesty and cheating pay off: the evolution of honest and dishonest equilibria in a conventional signalling game
Source: BMC Evol Biol. 2017 Dec 28;17:270. doi: 10.1186/s12862-017-1112-y (PMC5745956; doi:10.1186/s12862-017-1112-y)
Supplement: Supplementary file 4 — (PDF 728 kb) [file 12862_2017_1112_MOESM4_ESM.pdf]

Supplementary Information

When honesty and cheating pay off: the evolution of honest and dishonest equilibria in a conventional signalling game

Szabolcs Számadó

MTA TK "Lendület" Research Center for Educational and Network Studies (RECENS)

Hungary, Budapest, Tóth Kálmán u. 4. H-1097

Supplementary Tables

SI Table 1. Investigated scenarios. SS09: Szalai and Számadó (2009) pay-offs, H13: Helgesen et al. (2013) pay-offs; 36S and 8S: random and 8 strategy seedings respectively.

**Evolvability**		
Paramater set	Pay-offs	Initial strategies
SS09_code3	SS09	36S
	SS09	8S
	H13	36S
	H13	8S
SS09_code5	SS09	36S
	SS09	8S
	H13	36S
	H13	8S
SS09_code11	SS09	36S
	SS09	8S
	H13	36S
	H13	8S
SS09_code13	SS09	36S
	SS09	8S
	H13	36S
	H13	8S
SS09_code15	SS09	36S
	SS09	8S
	H13	36S
	H13	8S
SS09_code27	SS09	36S
	SS09	8S
	H13	36S
	H13	8S
H13	SS09	36S
	SS09	8S
	H13	36S
	H13	8S

SI Table 2. Evolvability: observed frequencies of no-signalling, honest and cheating outcomes.

runs	no-signalling	honesty	cheating
c3_36S_SS09	3119	783	1098
c3_36S_H13	3899	322	779
c3_8S_SS09	810	3379	811
c3_8S_H13	1375	3284	341
c5_36S_SS09	3500	416	1084
c5_36S_H13	3808	234	958
c5_8S_SS09	1823	2239	938
c5_8S_H13	1819	2624	557
c11_36S_SS09	3665	720	615
c11_36S_H13	4172	314	514
c11_8S_SS09	2560	1706	734
c11_8S_H13	3742	697	561
c13_36S_SS09	3406	153	1441
c13_36S_H13	3996	44	960
c13_8S_SS09	409	994	3597
c13_8S_H13	2406	395	2199
c15_36S_SS09	2707	115	2178
c15_36S_H13	3777	46	1177
c15_8S_SS09	1165	337	3498
c15_8S_H13	2813	173	2014
c27_36S_SS09	352	42	4606
c27_36S_H13	4807	4	189
c27_8S_SS09	193	52	4755
c27_8S_H13	4872	14	114
H13_36S_SS09	3330	0	270
H13_36S_H13	3377	0	223
H13_8S_SS09	3318	1	281
H13_8S_H13	3362	2	236

SI Table 3. The observed number of major strategy combinations, found in more than 0.01% of the runs (out of 30000 runs), as a function of different seedings and pay-offs when investigating the evolvability of polymorphic equilibria using the SS09 parameter range.Avg and sd denote the average frequency and standard deviation of the strategies supporting the given strategy combination.

**Seed: 36 strategies, Pay-offs: SS09**														
combination	observed	avg	±sd	avg	±sd	avg	±sd	avg	±sd	avg	±sd	avg	±sd
**{0, 9}**	5194	47,88	±16.99	48,06	±16.94								
**{22, 31}**	2943	47,82	±17.22	48,24	±17.27								
**{0, 9, 18, 27}**	740	25,81	±11.21	26,59	±11.31	19,83	±10.09	19,96	±10.23				
**{1, 15}**	713	60,81	±10.67	30,07	±9.15								
**{4, 7, 16, 17, 25, 35}**	557	20,53	±5.05	20,23	±3.59	15,16	±3.78	16,37	±3.57	10,97	±3.11	9,19	±2.71
**{17, 19, 34, 35}**	544	16,45	±8.0	40,26	±7.94	27,97	±4.48	9,02	±3.11				
**{31}**	503	87,31	±4.38										
**{5, 8, 13, 14, 26, 32}**	500	15,2	±3.91	16,06	±3.42	20,5	±4.99	20,32	±3.65	9,29	±2.75	11,16	±3.18
**{2, 12}**	496	29,15	±9.21	61,75	±11.06								
**{1, 7, 15, 17}**	482	44,44	±9.3	14,66	±5.92	23,47	±7.57	10,32	±4.06				
**{22}**	479	86,82	±4.8										
**{1, 7, 15}**	451	60,37	±9.13	13,13	±5.54	17,2	±9.01						
**{8, 23, 26, 30}**	420	15,71	±8.38	27,94	±4.42	8,7	±2.98	41,32	±8.47				
**{17, 19, 34}**	412	15,98	±12.95	46,57	±9.64	28,65	±4.73						
**{2, 8, 12, 14}**	361	22,55	±7.41	9,91	±3.85	45,02	±9.23	15,63	±5.95				
**{2, 12, 14}**	348	16,95	±9.22	61,16	±9.28	12,98	±5.29						
**{8, 23, 30}**	341	16,09	±13.16	28,82	±4.55	46,31	±9.85						
**{23, 30}**	304	29,91	±3.11	58,65	±3.89								

Seed: 8 strategies, Pay-offs: SS09													
combination	observed	avg	±sd	avg	±sd	avg	±sd	avg	±sd	avg	±sd	avg	±sd
**{0, 9}**	2610	48,12	±18.33	48,86	±18.38								
**{2, 30}**	2566	53,81	±12.18	39,58	±11.0								
**{2, 12}**	2204	27,15	±10.13	64,65	±12.31								
**{20, 30}**	2003	28,7	±6.46	59,04	±7.72								
**{18, 27}**	1539	46,59	±12.56	45,66	±12.59								
**{2, 12, 20, 30}**	1123	15,83	±6.71	29,75	±11.67	13,21	±5.42	31,12	±11.67				
**{2, 8, 12, 14}**	1000	24,47	±7.43	10,7	±4.15	42,5	±9.1	15,06	±6.01				
**{19, 30}**	918	47,2	±4.8	47,2	±4.72								
**{23, 30}**	888	28,72	±3.62	59,67	±3.83								
**{5, 8, 13, 14, 26, 32}**	851	15,22	±3.84	16,15	±3.43	20,35	±5.17	20,27	±3.53	9,35	±2.69	11,12	±3.17
**{14, 23, 30}**	836	13,19	±5.26	18,55	±4.92	54,69	±5.35						
**{0, 9, 18, 27}**	721	22,59	±11.37	23,16	±11.26	23,88	±12.45	24,13	±12.22				
**{2, 12, 14}**	703	17,6	±9.97	60,46	±10.4	12,86	±5.48						
**{22, 31}**	644	48,48	±16.63	47,66	±16.52								
**{8, 23, 26, 30}**	483	15,14	±8.13	27,49	±4.27	8,64	±3.08	41,21	±8.4				
**{5, 8, 13, 14, 32}**	478	15,26	±4.39	13,66	±4.69	28,88	±7.78	23,49	±3.65	9,46	±2.96		
**{20, 27, 30}**	408	25,59	±5.77	8,09	±2.43	55,88	±7.25						
**{2, 12, 30}**	392	24,07	±10.64	43,76	±13.79	20,01	±10.43						
**{14, 23, 30, 32}**	387	13,15	±5.39	15,27	±4.42	50,55	±5.95	9,36	±3.03				
**{2, 14, 30}**	375	25,75	±10.44	12,73	±5.17	50,53	±7.88						
**{8, 14, 23, 30}**	345	8,03	±2.28	13,56	±4.93	20,92	±4.4	45,95	±4.67				
**{5, 8, 13, 14}**	316	16,95	±4.59	14,14	±5.53	34,23	±9.69	25,53	±3.84				
**{8, 23, 30}**	311	11,63	±8.23	29,21	±3.83	49,42	±7.2						

Seed: 36 strategies, Pay-offs: H13									
combination	observed	avg	±sd	avg	±sd	avg	±sd	avg	±sd
**{0, 9}**	4206	48,58	&17.65	48,2	&17.66				
**{22, 31}**	2519	48,44	&15.43	48,14	&15.43				
**{0, 9, 18, 27}**	535	27,3	&10.98	26,13	&10.98	20,13	&10.44	20,02	&9.92
**{7, 8}**	471	23,34	&16.8	66,09	&17.16				
**{14, 17}**	460	24,18	&17.82	65,6	&18.18				
**{17, 19, 34, 35}**	336	20,99	&12.49	34,89	&9.77	23,81	&5.17	15,19	&8.85
**{8, 23, 26, 30}**	334	21,29	&13.46	23,85	&5.36	14,64	&9.16	35,27	&10.45
**{21, 22, 23, 25}**	301	13,89	&7.12	50,8	&13.86	12,23	&5.78	13,9	&6.74

Seed: 8 strategies, Pay-offs: H13									
Combination	observed	avg	±sd	avg	±sd	avg	±sd	avg	±sd
**{20, 30}**	3038	30,39	±6.18	62,21	±6.25				
**{0, 9}**	2410	48,42	±22.91	49,34	±22.88				
**{2, 30}**	2059	54,47	±12.39	40,21	±11.14				
**{18, 27}**	1723	48,03	±14.42	49,1	±14.39				
**{2, 12}**	841	20,2	±8.32	74,18	±8.02				
**{19, 30}**	778	48,34	±3.41	47,95	±3.31				
**{0, 9, 18, 27}**	740	23,09	±10.46	22,16	±11.01	26,02	±12.79	24,8	±12.83
**{2, 12, 20, 30}**	674	12,8	±5.1	29,98	±11.35	14,69	±5.61	35,34	±10.06
**{14, 17}**	562	34,25	±22.27	56,43	±22.51				
**{7, 8}**	528	34,97	±21.75	55,68	±22.18				
**{22, 31}**	408	49,49	±16.44	47,8	±16.73				

SI Table 4. The observed number of major strategy combinations, found in more than 0.01% of the runs (out of 3600 runs), as a function of different seedings and pay-offs when investigating the evolvability of polymorphic equilibria using the H13 parameter range. Avg and sd denote the average frequency and standard deviation of the strategies supporting the given strategy combination.

MC_summary_Helgesen_random_RSS09_SS09 Seed: 36 strategies, Pay-offs: SS09

combination	observed	avg	±sd	avg	±sd	avg	±sd
**{22, 31}**	2205	47,92	±20.32	47,45	±20.32		
**{0, 9}**	458	46,53	±12.29	45,32	±12.36		
**{22}**	59	92,61	±2.75				
**{31}**	48	92,88	±2.42				
**{0, 1, 9}**	42	51,33	±10.95	7,6	±2.12	35,07	±11.69
**{0, 9, 12}**	39	31,21	±8.18	54,77	±8.11	8,13	±2.3

MC_summary_Helgesen_eightS_RSS09_SS09 Seed: 8 strategies, Pay-offs: SS09

combination	observed	avg	±sd	avg	±sd	avg	±sd
**{22, 31}**	2201	48,41	±20.07	47,01	±20.16		
**{0, 9}**	489	46,02	±11.83	45,84	±11.75		
**{22}**	49	92,18	±2.56				
**{31}**	42	93,24	±2.54				
**{0, 1, 9}**	42	55,74	±10.41	8,14	±2.39	29,98	±9.57

MC_summary_Helgesen_random_RSS09_E85 Seed: 36 strategies, Pay-offs: H13

combination	observed	avg	±sd	avg	±sd	avg	±sd
**{22, 31}**	2023	47,13	±20.56	47,23	±20.52		
**{0, 9}**	473	45,91	±12.23	45,78	±12.38		
**{31}**	102	90,3	±3.12				
**{22}**	99	89,93	±3.14				
**{0, 9, 12}**	41	29,8	±11.33	55,66	±10.11	8,37	±3.01
**{0, 1, 9}**	37	53,46	±11.74	8,11	±3.01	32,46	±12.34

MC_summary_Helgesen_eightS_RSS09_E85 Seed: 8 strategies, Pay-offs: H13

combination	observed	avg	±sd	avg	±sd	avg	±sd
**{22, 31}**	1997	47,5	±20.41	46,98	±20.42		
**{0, 9}**	484	45,54	±12.68	45,97	±12.85		
**{22}**	93	90,54	±3.38				
**{31}**	90	90,14	±2.94				
**{0, 1, 9}**	47	55,06	±10.24	7,87	±2.11	30,77	±10.25
**{0, 9, 12}**	42	33,24	±9.34	52,95	±9.13	7,43	±1.64
