# Supplementary material for: When honesty and cheating pay off: the evolution of honest and dishonest equilibria in a conventional signalling game
Source: BMC Evol Biol. 2017 Dec 28;17:270. doi: 10.1186/s12862-017-1112-y (PMC5745956; doi:10.1186/s12862-017-1112-y)

Supplementary Information

When honesty and cheating pay off: the evolution of honest and dishonest equilibria in a conventional signalling game

Szabolcs Számadó

MTA TK "Lendület" Research Center for Educational and Network Studies (RECENS)

Hungary, Budapest, Tóth Kálmán u. 4. H-1097

Supplementary figures – H13 range

Figures 1-4 show the timelines of 20 independent individual runs for a given parameter combination from the H13 parameter range, even and odd numbered figures using the H13 and the SS09 pay-offs respectively, where the first ten runs in each figure (a) are seeded with 8 strategies used in the SS09 study while the second set of 10 runs (b) seeded with random mix of 36 strategies.

Figure 1. Individual timelines with the Helgesen et al (2013) parameters, pay-offs: H13. Each figure shows ten independent runs with the same parameter combination; (a) 8 strategy seed, (b) 36 strategy seed. Strategy codes are displayed on the right. $V = 100.0$, $Css = 15.0$, $Cww = 15.0$, $Cws = 50.0$, $Csw = 15.0$, $Ff = 0$.

(a)

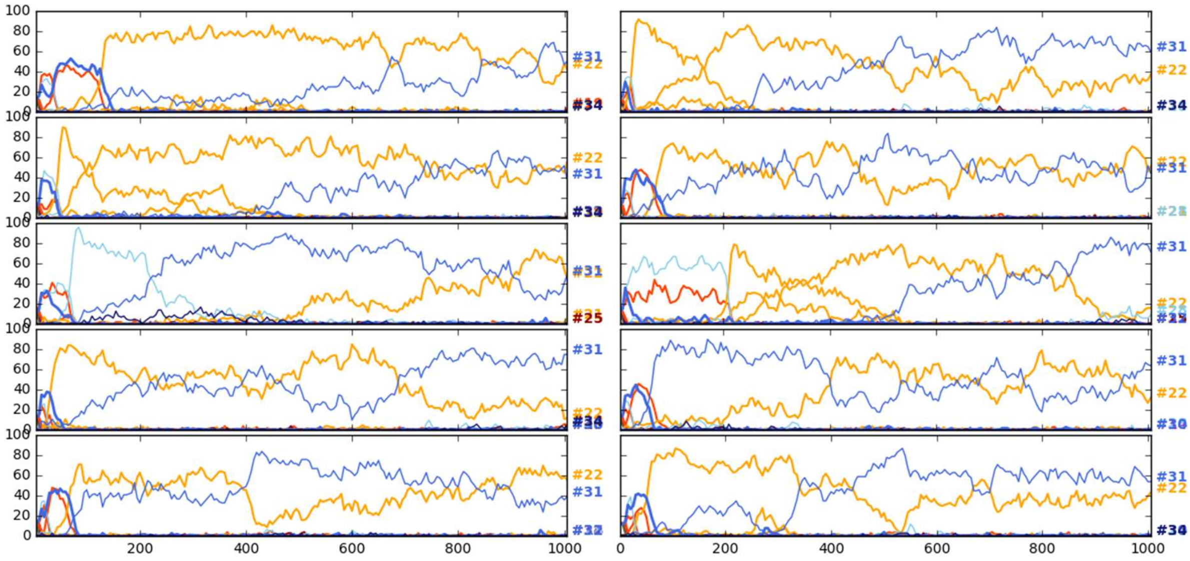

(b)

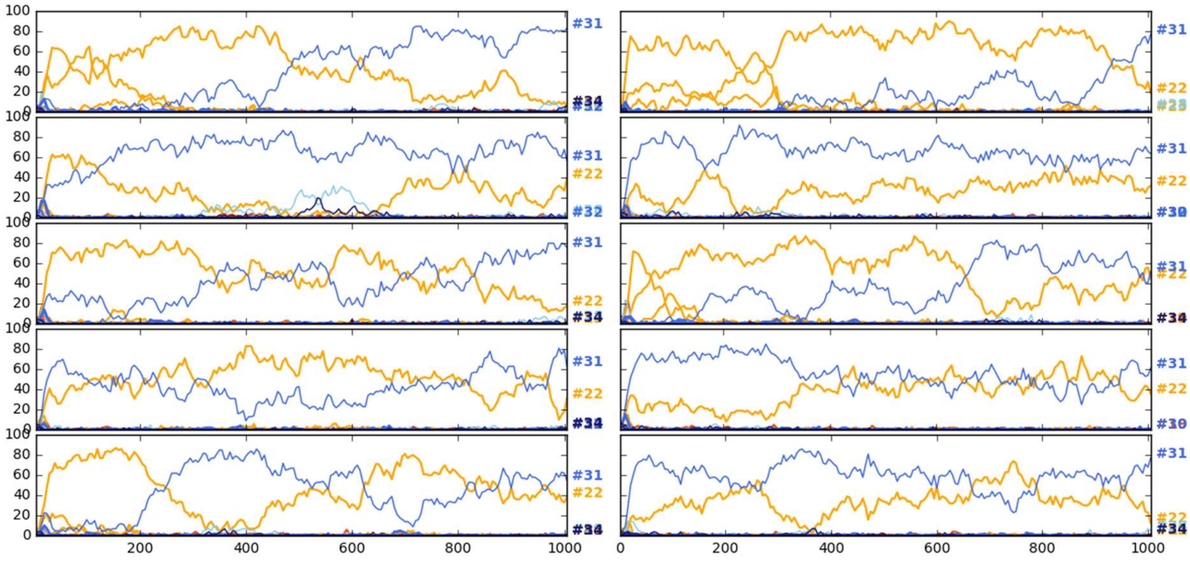

Figure 2. Individual timelines with the Helgesen et al (2013) parameters, pay-offs: SS09. Each figure shows ten independent runs with the same parameter combination; (a) 8 strategy seed, (b) 36 strategy seed. Strategy codes are displayed on the right. *V* = 100.0, *Css* = 15.0, *Cww* = 15.0, *Cws* = 50.0, *Csw* = 15.0, *Ff* = 0.

(a)

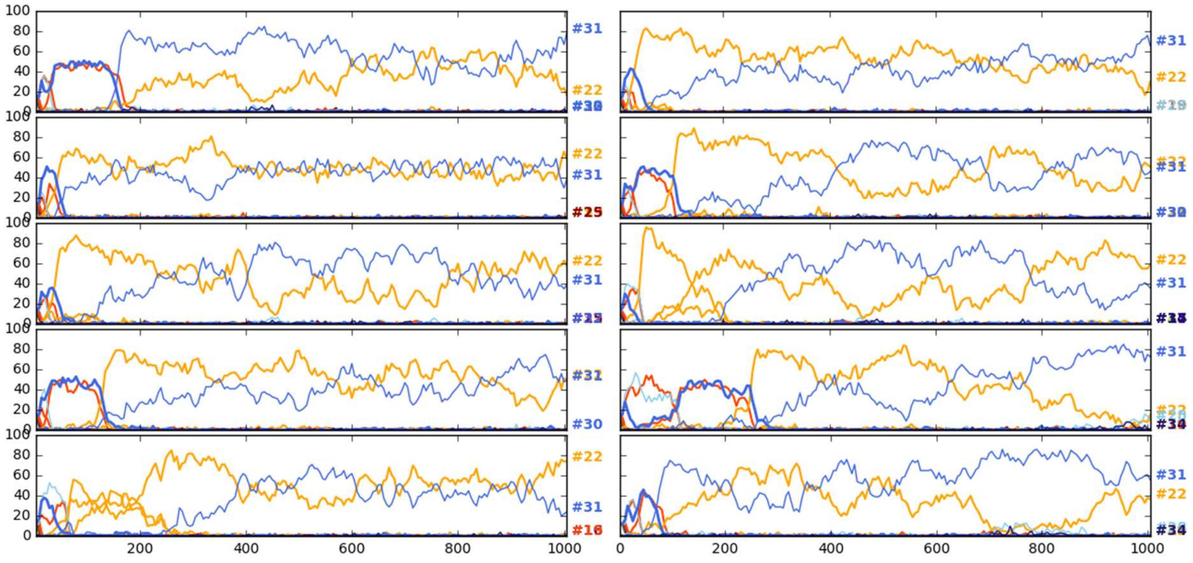

(b)

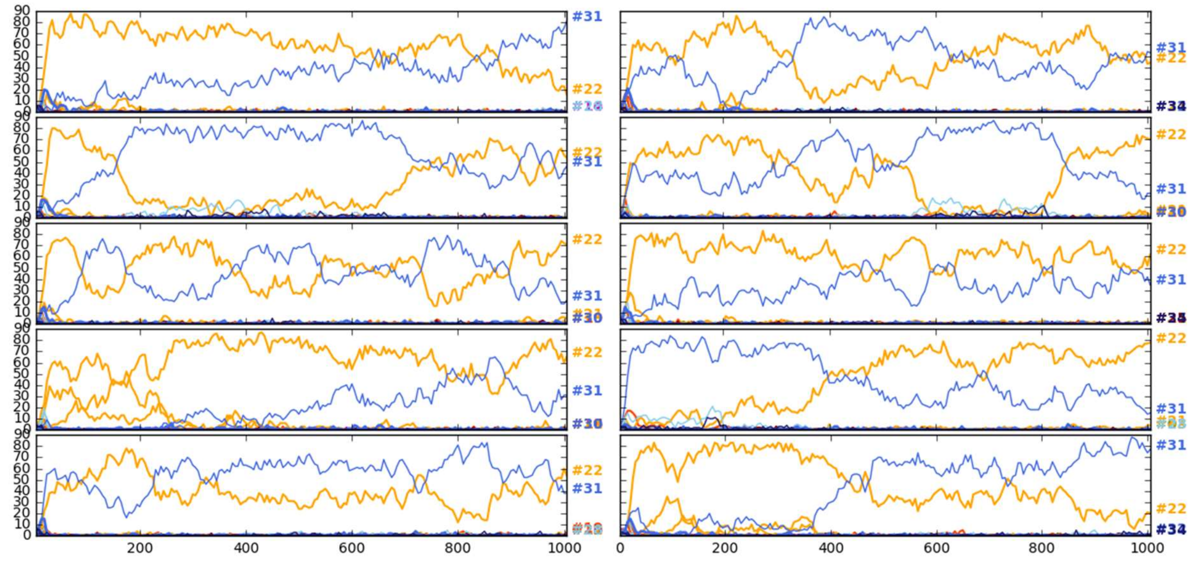

Figure 3. Individual timelines with the Helgesen et al (2013) parameters, pay-offs: H13. Each figure shows ten independent runs with the same parameter combination; (a) 8 strategy seed, (b) 36 strategy seed. Strategy codes are displayed on the right. *V* = 100.0, *Css* = 15.0, *Cww* = 15.0, *Cws* = 70.0, *Csw* = 15.0, *Ff* = 0.

(a)

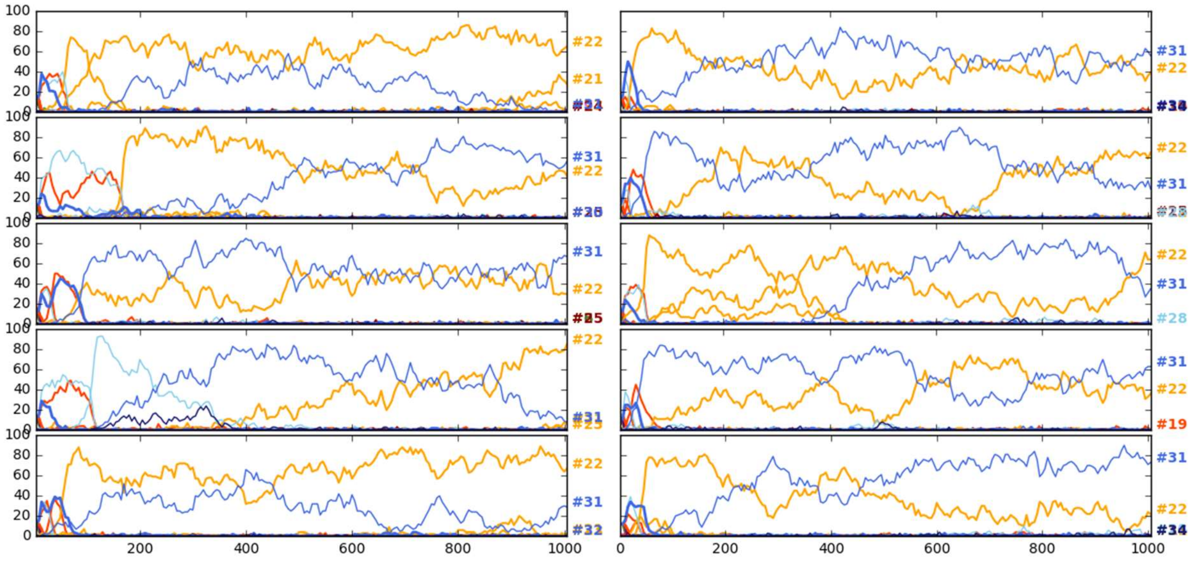

(b)

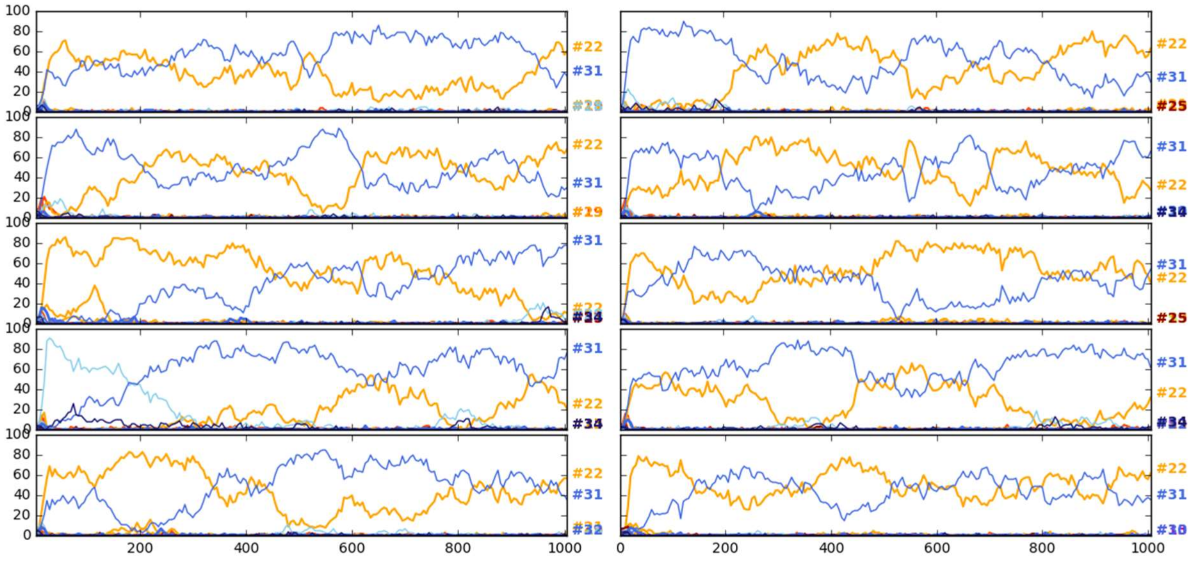

Figure 4. Individual timelines with the Helgesen et al (2013) parameters, pay-offs: SS09. Each figure shows ten independent runs with the same parameter combination; (a) 8 strategy seed, (b) 36 strategy seed. Strategy codes are displayed on the right. V = 100.0, Css = 15.0, Cww = 15.0, Cws = 70.0, Csw = 15.0, Ff = 0.

(a)

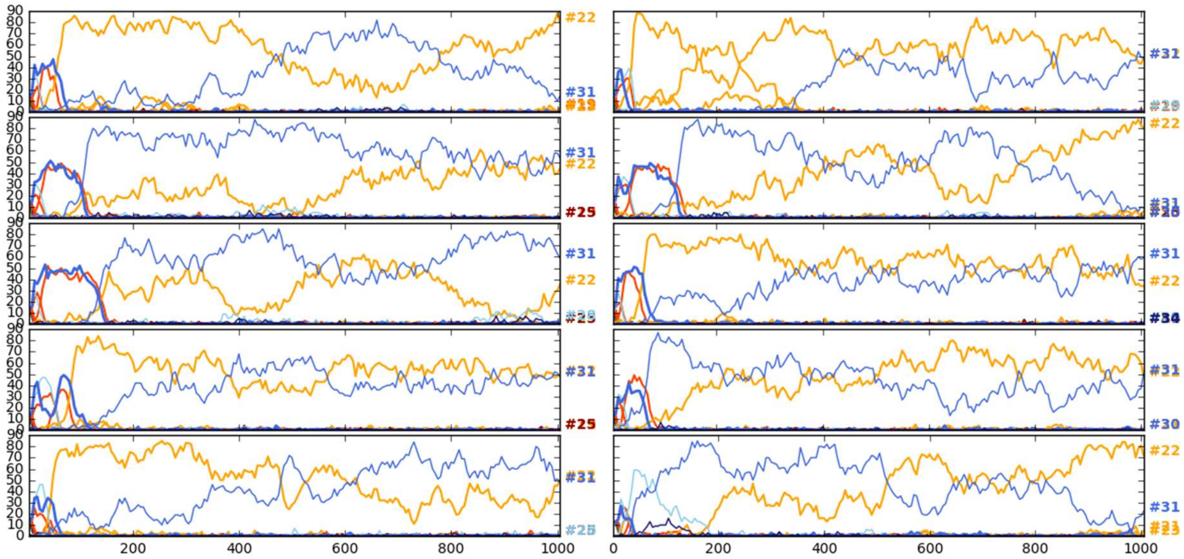

(b)

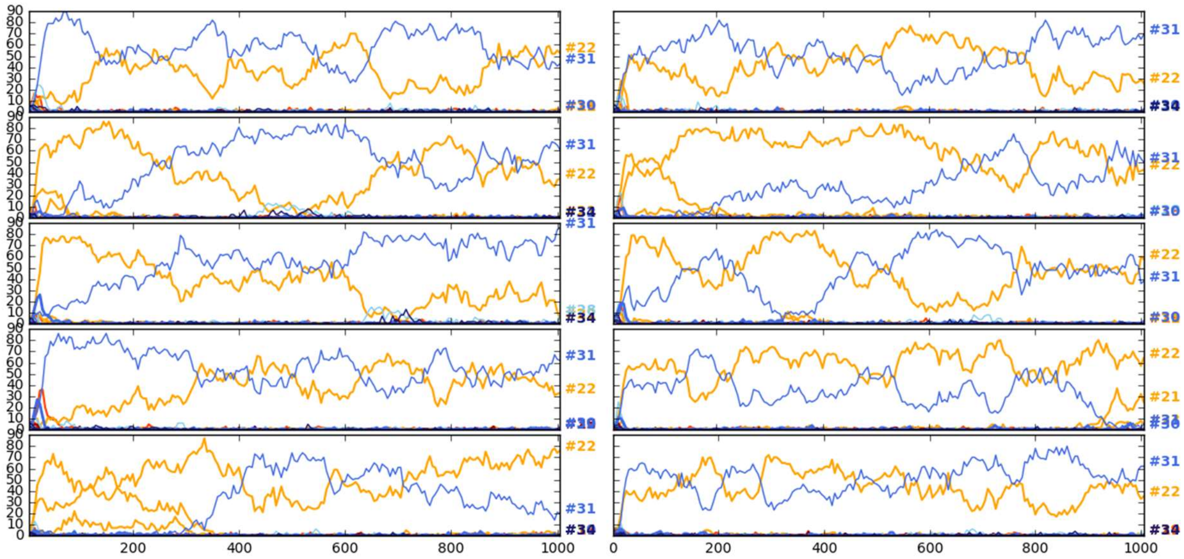

Supplement: Supplementary file 5 — Individual timelines SS09 parameter set. (PDF 1820 kb) [file 12862_2017_1112_MOESM5_ESM.pdf]
