# Supplementary material for: When honesty and cheating pay off: the evolution of honest and dishonest equilibria in a conventional signalling game
Source: BMC Evol Biol. 2017 Dec 28;17:270. doi: 10.1186/s12862-017-1112-y (PMC5745956; doi:10.1186/s12862-017-1112-y)

Supplementary Information

When honesty and cheating pay off: the evolution of honest and dishonest equilibria in a conventional signalling game

Szabolcs Számadó

MTA TK "Lendület" Research Center for Educational and Network Studies (RECENS)

Hungary, Budapest, Tóth Kálmán u. 4. H-1097

Supplementary figures – SS09 range

Figures 1-24 show the timelines of 20 independent individual runs for a given parameter combination from the SS09 parameter range, even and odd numbered figures using the H13 and the SS09 pay-offs respectively, where the first ten runs in each figure (a) are seeded with 8 strategies used in SS09 while the second set of 10 runs (b) seeded with random mix of 36 strategies.

Figure 1. Timelines of the parameter combination: 639 (parameter combinations are defined in SI file 2). Pay-offs: H13; seed: eight str.; parameter region: code3. Each figure shows ten independent runs with the same parameter combination: (a) 8 strategy seed, (b) 36 strategy seed. Strategy codes are displayed on the right. *V* = 10.0, *Css* = 35.0, *Cww* = 17.0, *Cws* = 35.0, *Csw* = 27.0, *Ff* = 2.0.

(a)

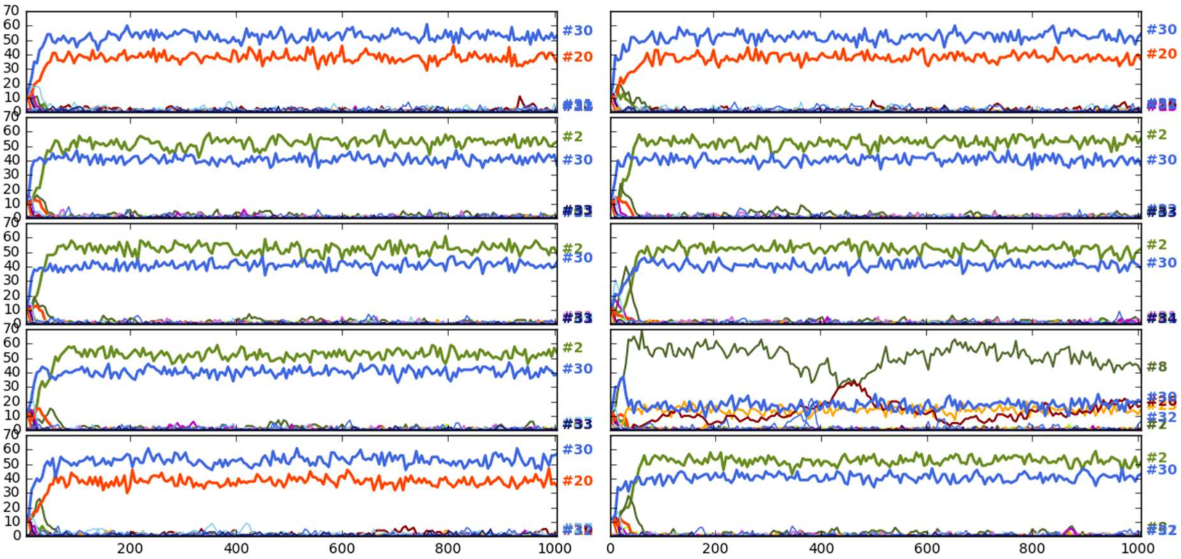

(b)

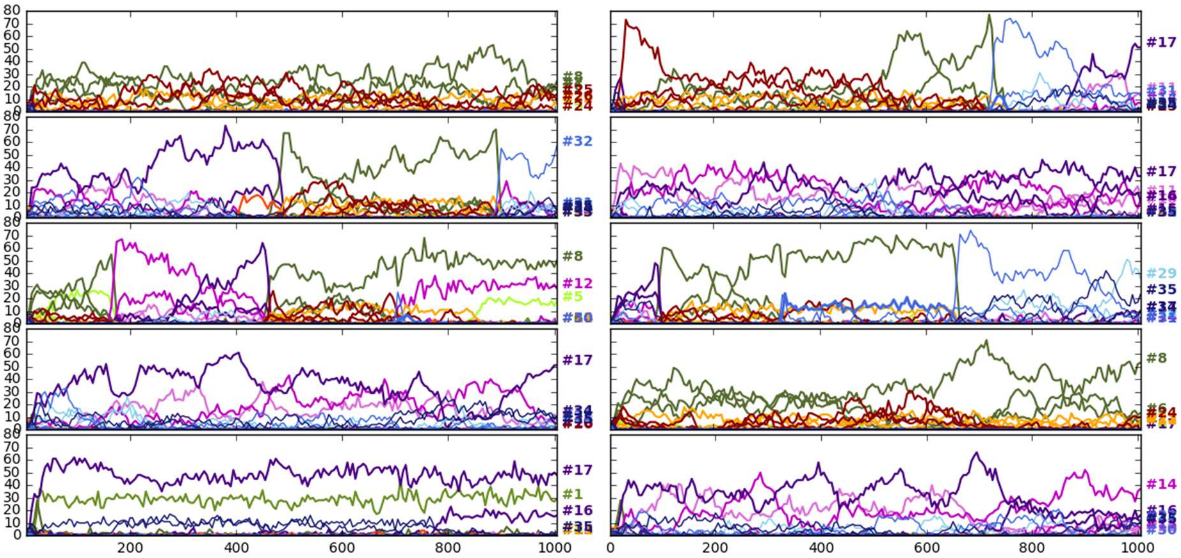

Figure 2. Timelines of the parameter combination: 639 (parameter combinations are defined in SI file 2). Pay-offs: SS09; seed: eight str.; parameter region: code3. Each figure shows ten independent runs with the same parameter combination: (a) 8 strategy seed, (b) 36 strategy seed. Strategy codes are displayed on the right. *V* = 10.0, *Css* = 35.0, *Cww* = 17.0, *Cws* = 35.0, *Csw* = 27.0, *Ff* = 2.0.

(a)

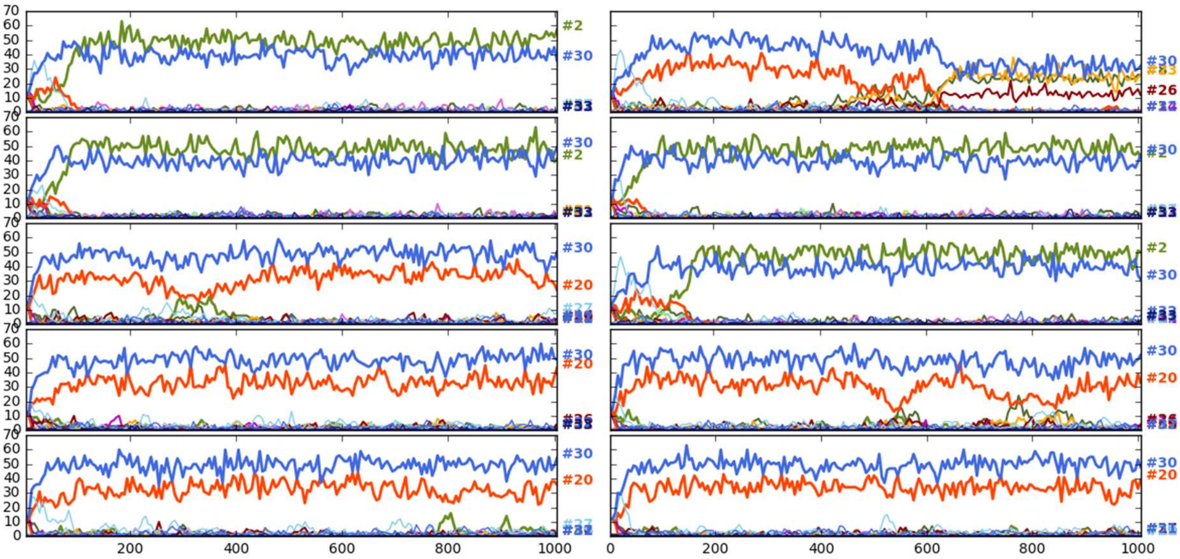

(b)

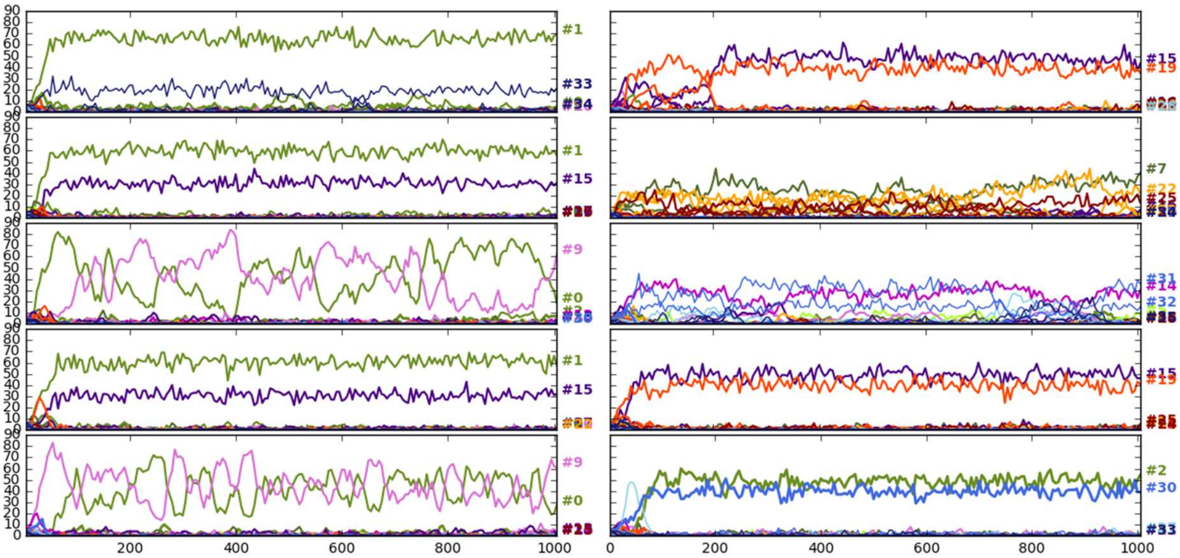

Figure 3. Timelines of the parameter combination: 984. Pay-offs: H13; seed: eight str.; parameter region: code3; (a) 8 strategy seed, (b) 36 strategy seed. *V* = 13.0, *Css* = 20.0, *Cww* = 7.0, *Cws* = 15.0, *Csw* = 17.0, *Ff* = 0.0.

(a)

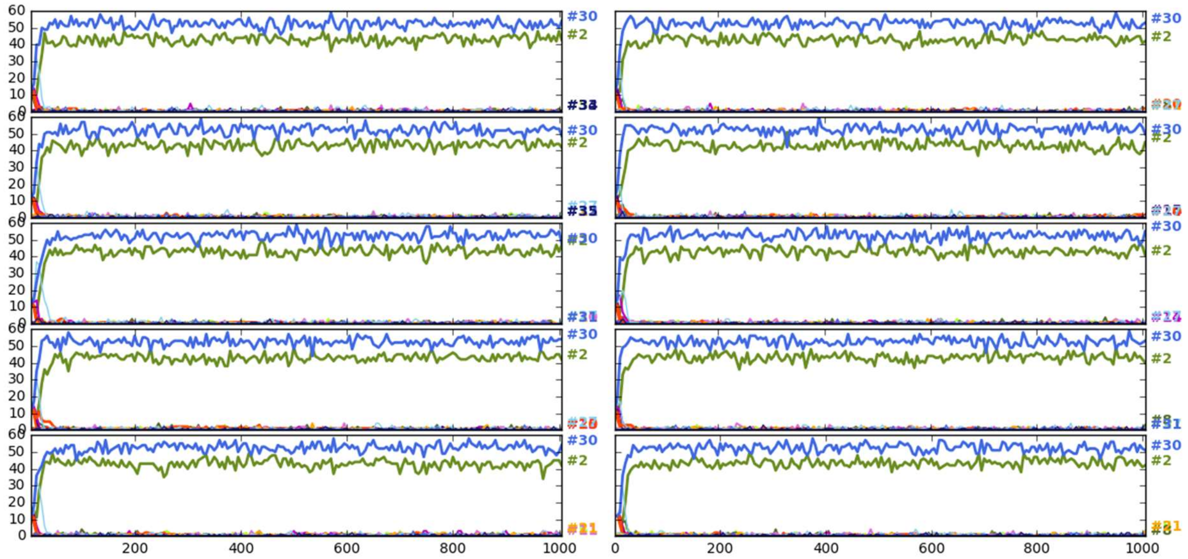

(b)

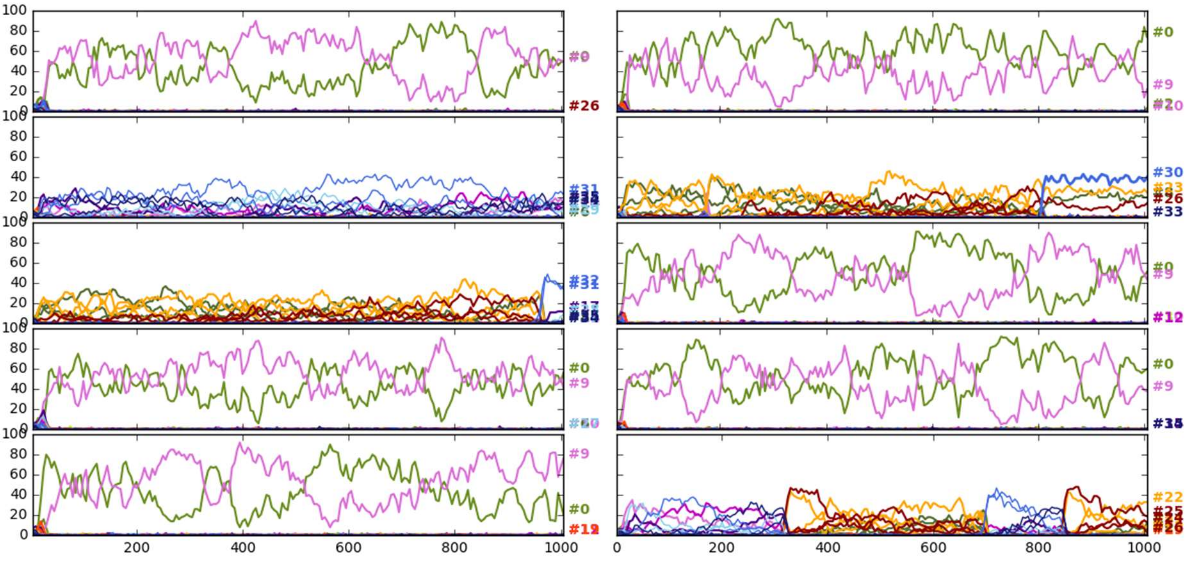

Figure 4. Timelines of the parameter combination: 984. Pay-offs: SS09; seed: eight str.; parameter region: code3; (a) 8 strategy seed, (b) 36 strategy seed. *V* = 13.0, *Css* = 20.0, *Cww* = 7.0, *Cws* = 15.0, *Csw* = 17.0, *Ff* = 0.0.

(a)

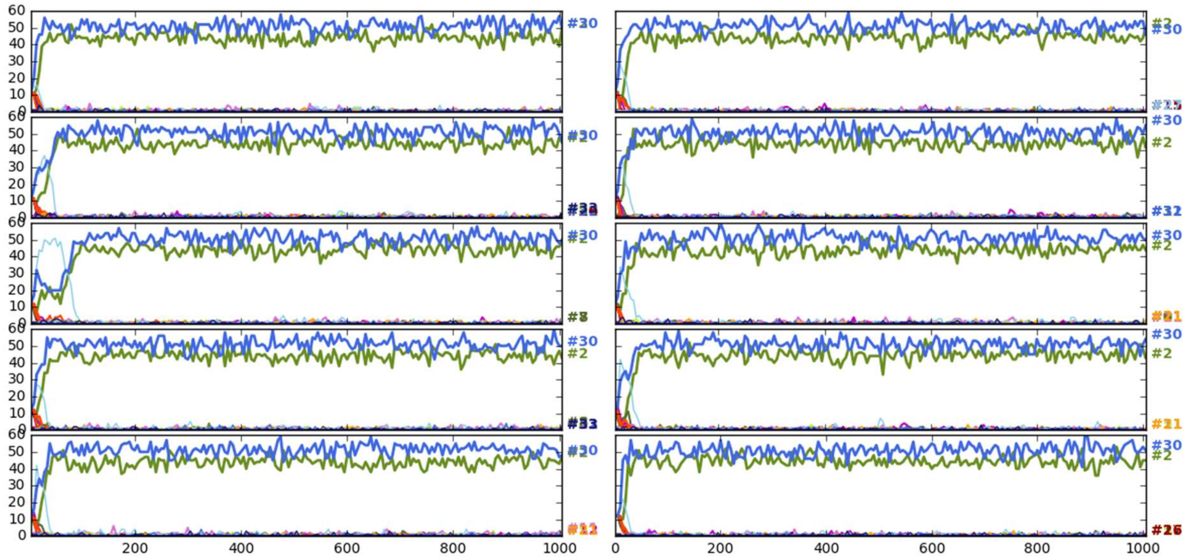

(b)

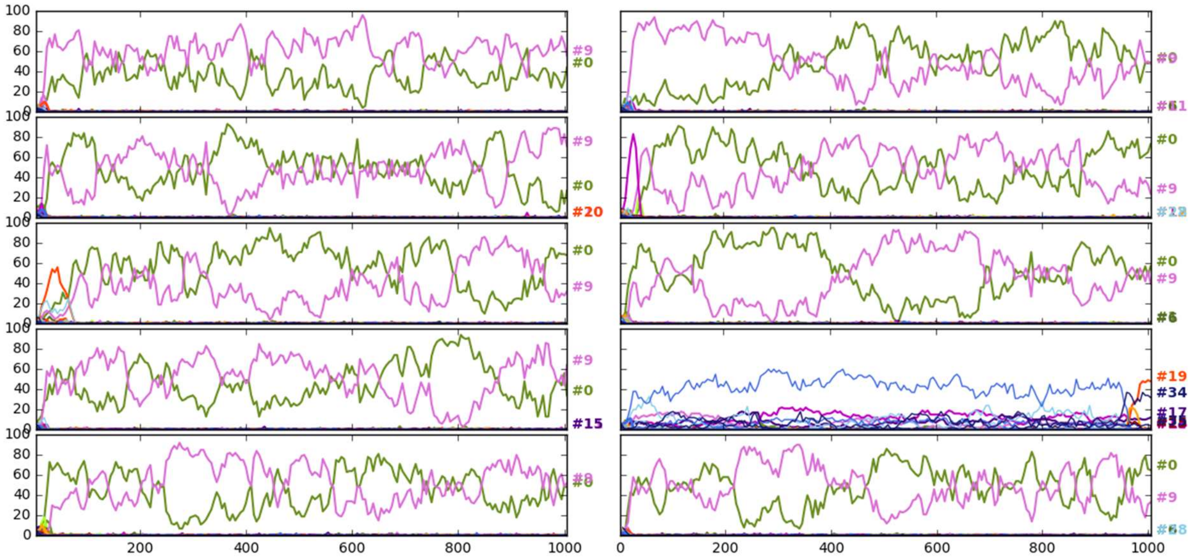

Figure 5. Timelines of the parameter combination: 6269. Pay-offs: H13; seed: eight str.; parameter region: code5; (a) 8 strategy seed, (b) 36 strategy seed. *V* = 34.0, *Css* = 30.0, *Cww* = 12.0, *Cws* = 30.0, *Csw* = 17.0, *Ff* = 0.0.

(a)

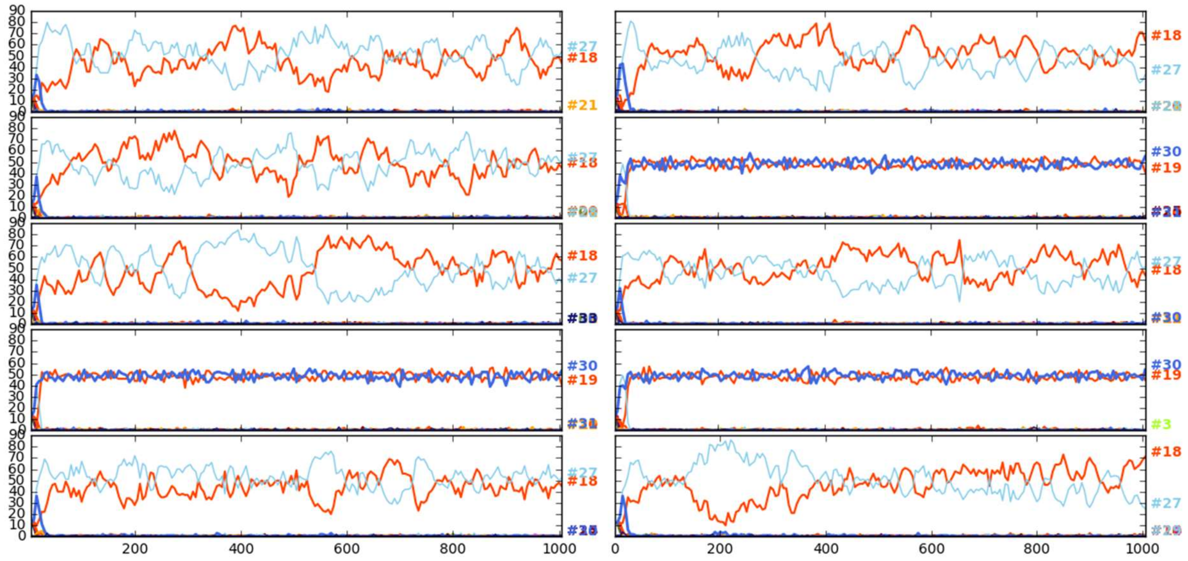

(b)

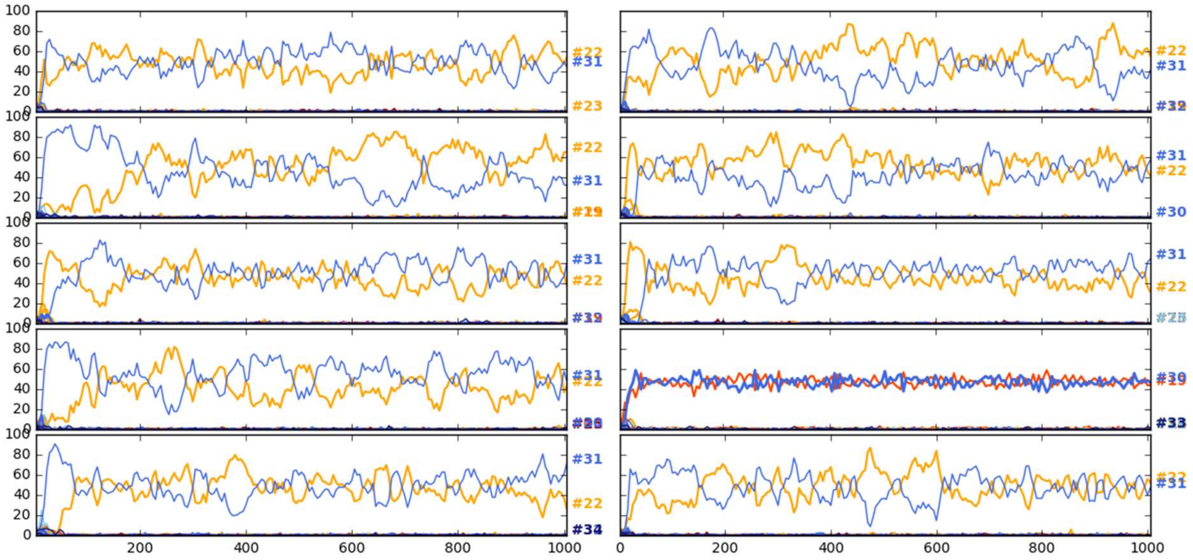

Figure 6. Timelines of the parameter combination: 6269. Pay-offs: SS09; seed: eight str.; parameter region: code5; (a) 8 strategy seed, (b) 36 strategy seed. *V* = 34.0, *Css* = 30.0, *Cww* = 12.0, *Cws* = 30.0, *Csw* = 17.0, *Ff* = 0.0.

(a)

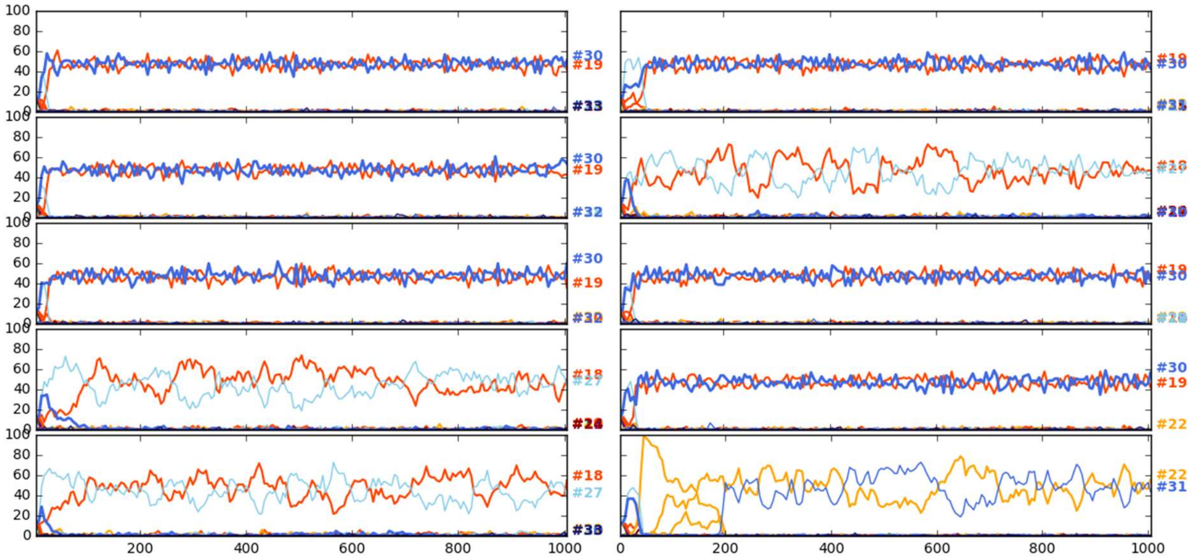

(b)

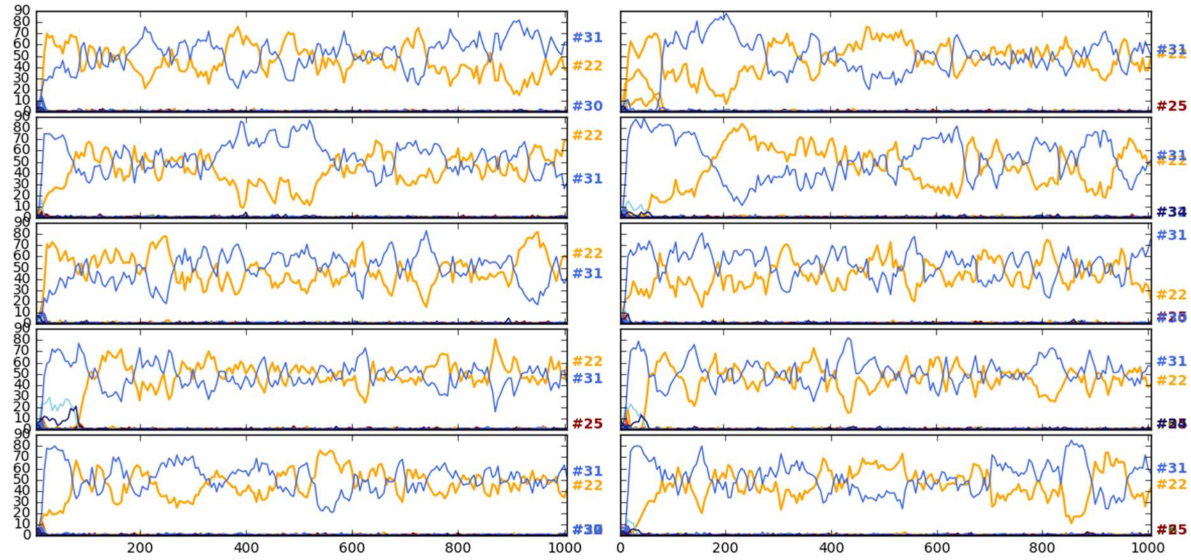

Figure 7. Timelines of the parameter combination: 600. Pay-offs: H13; seed: eight str.; parameter region: code5; (a) 8 strategy seed, (b) 36 strategy seed. *V* = 10.0, *Css* = 35.0, *Cww* = 17.0, *Cws* = 30.0, *Csw* = 17.0, *Ff* = 0.0.

(a)

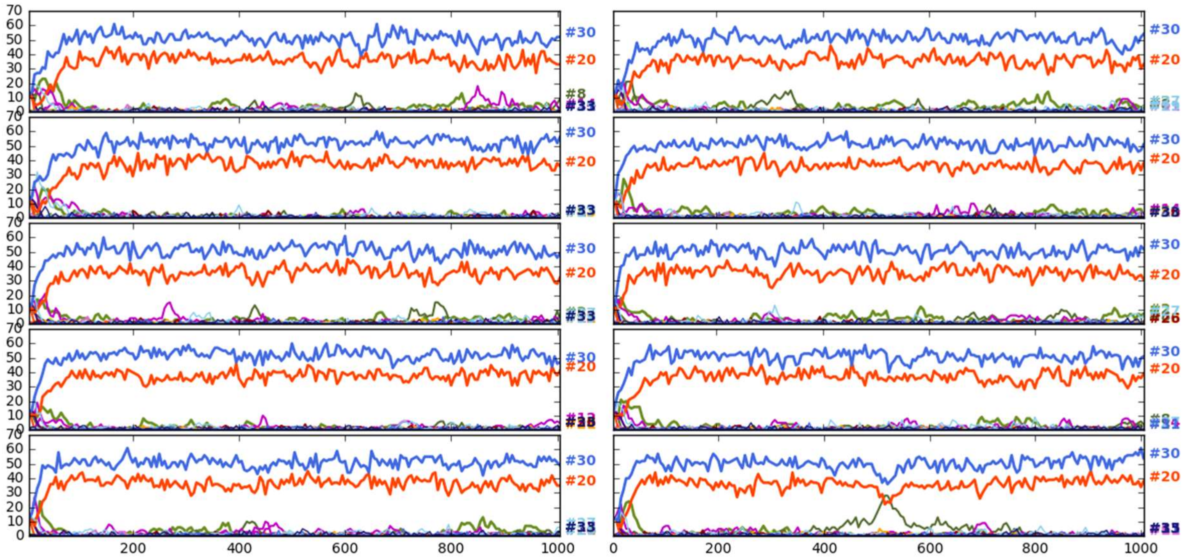

(b)

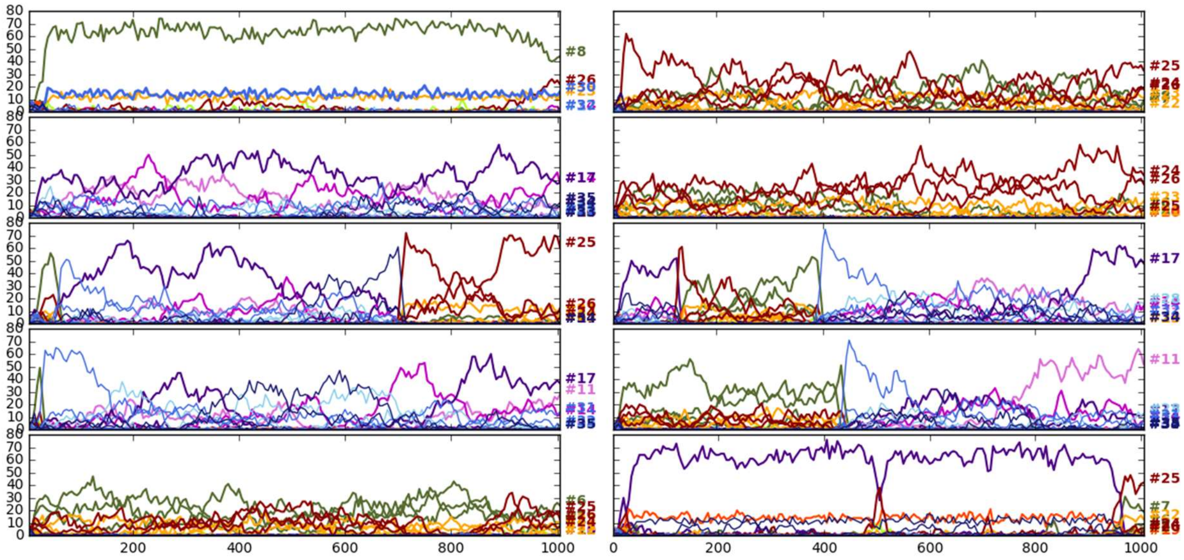

Figure 8. Timelines of the parameter combination: 600. Pay-offs: SS09; seed: eight str.; parameter region: code5; (a) 8 strategy seed, (b) 36 strategy seed. *V* = 10.0, *Css* = 35.0, *Cww* = 17.0, *Cws* = 30.0, *Csw* = 17.0, *Ff* = 0.0.

(a)

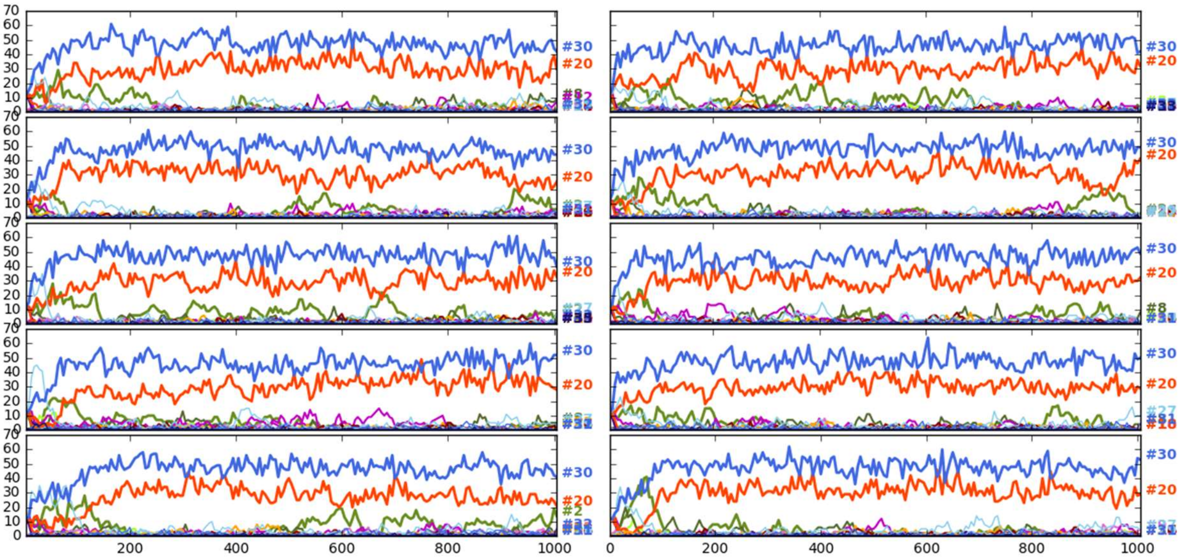

(b)

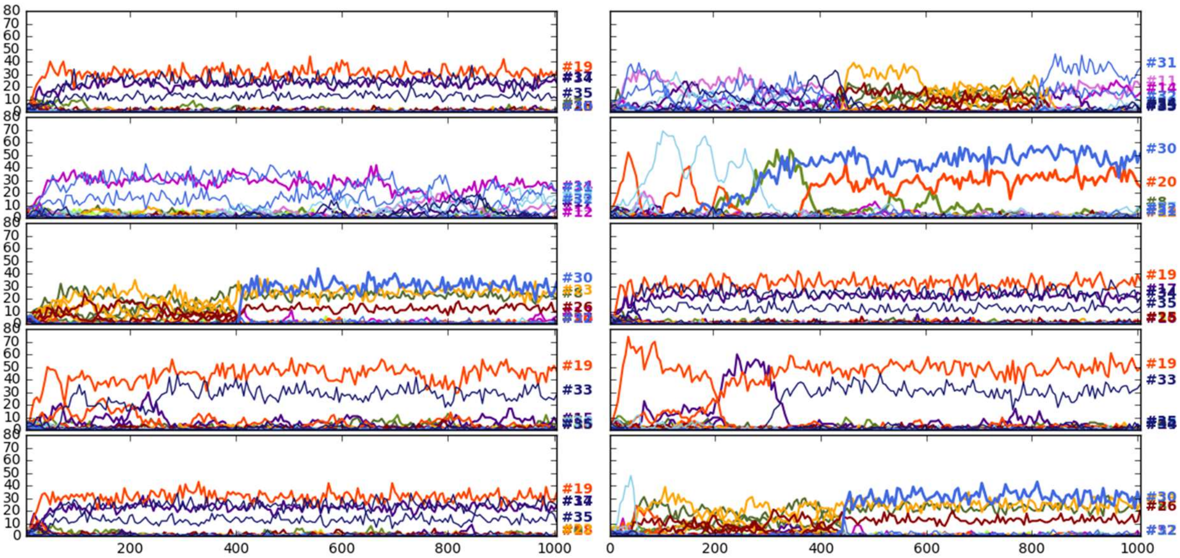

Figure 9. Timelines of the parameter combination: 4226. Pay-offs: H13; seed: eight str.; parameter region: code11; (a) 8 strategy seed, (b) 36 strategy seed. V = 22.0, Css = 35.0, Cww = 12.0, Cws = 30.0, Csw = 27.0, Ff = 9.0.

(a)

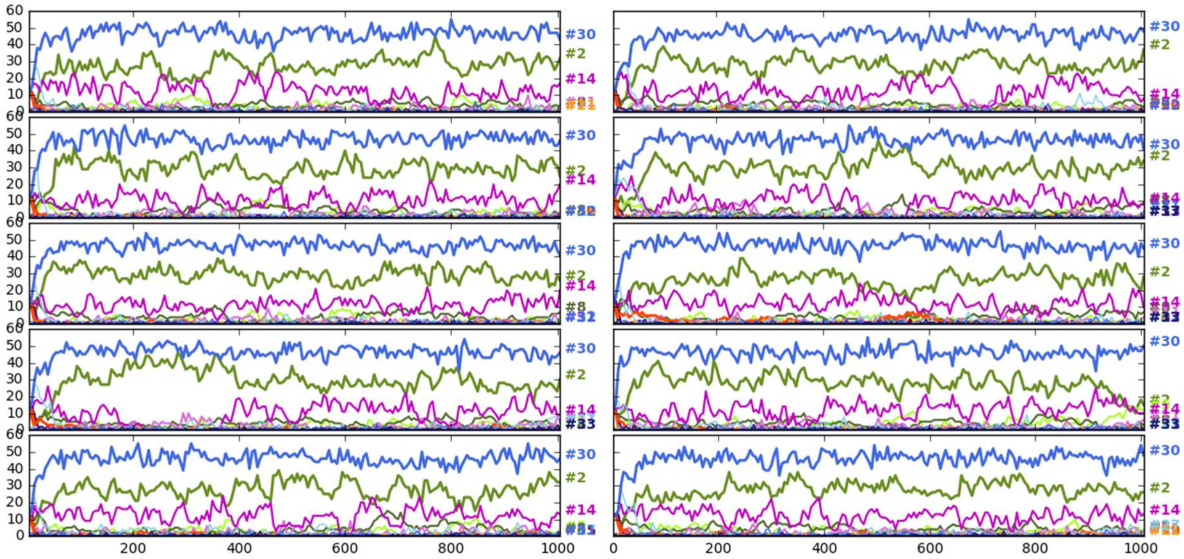

(b)

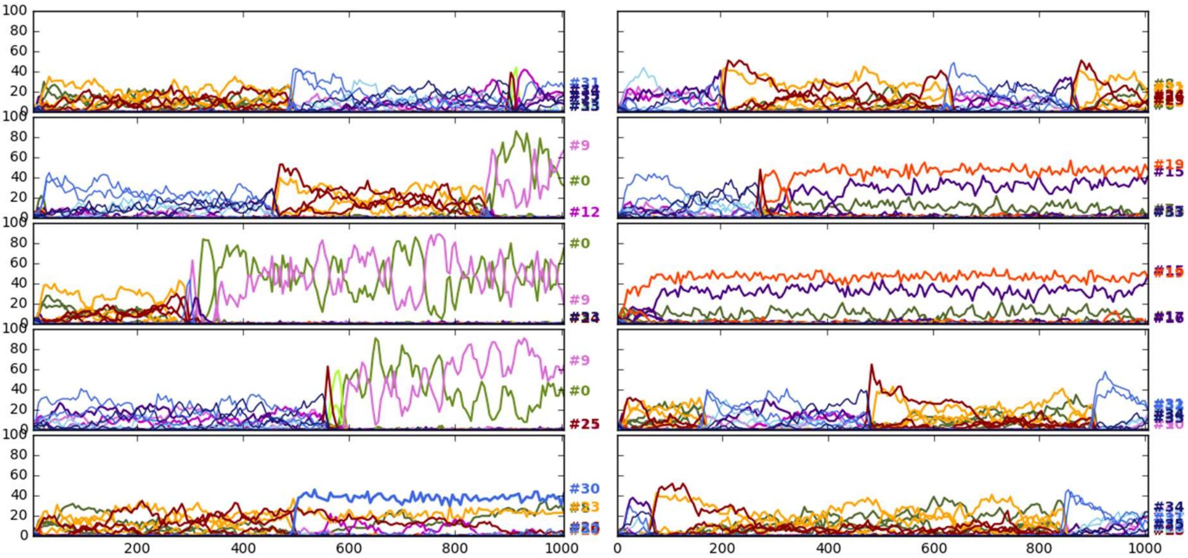

Figure 10. Timelines of the parameter combination: 4226. Pay-offs: SS09; seed: eight str.; parameter region: code11; (a) 8 strategy seed, (b) 36 strategy seed. *V* = 22.0, *Css* = 35.0, *Cww* = 12.0, *Cws* = 30.0, *Csw* = 27.0, *Ff* = 9.0.

(a)

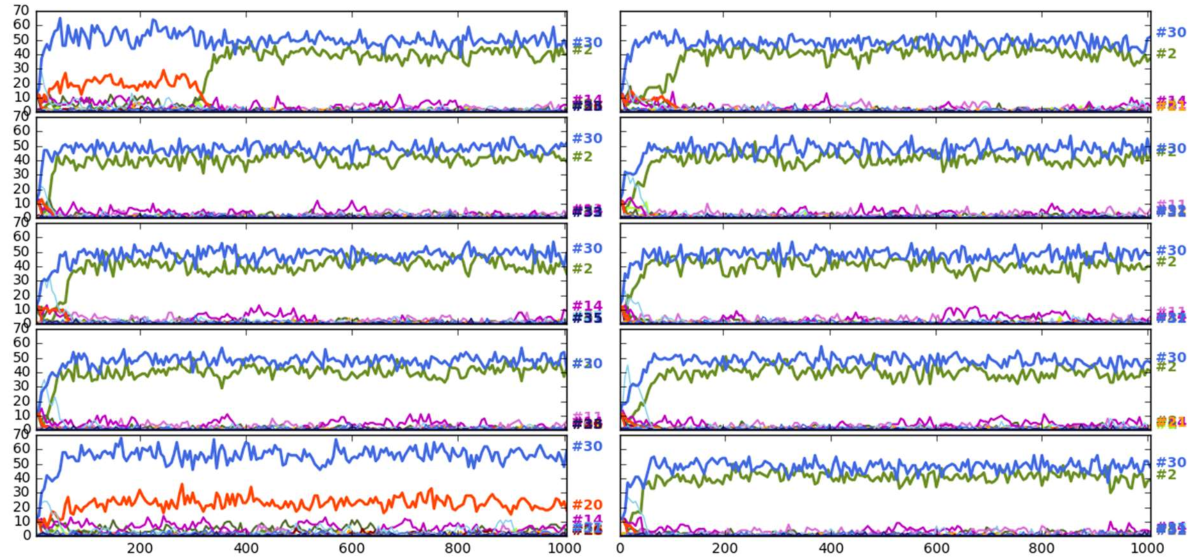

(b)

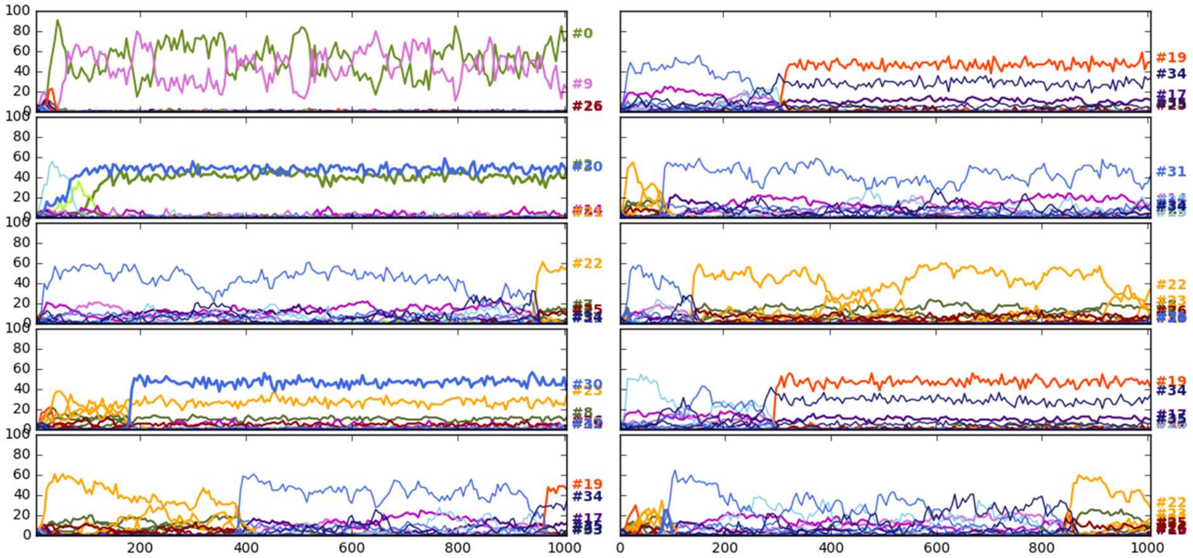

Figure 11. Timelines of the parameter combination: 3769. Pay-offs: H13; seed: eight str.; parameter region: code11; (a) 8 strategy seed, (b) 36 strategy seed. *V* = 1.0, *Css* = 35.0, *Cww* = 7.0, *Cws* = 15.0, *Csw* = 32.0, *Ff* = 0.0.

(a)

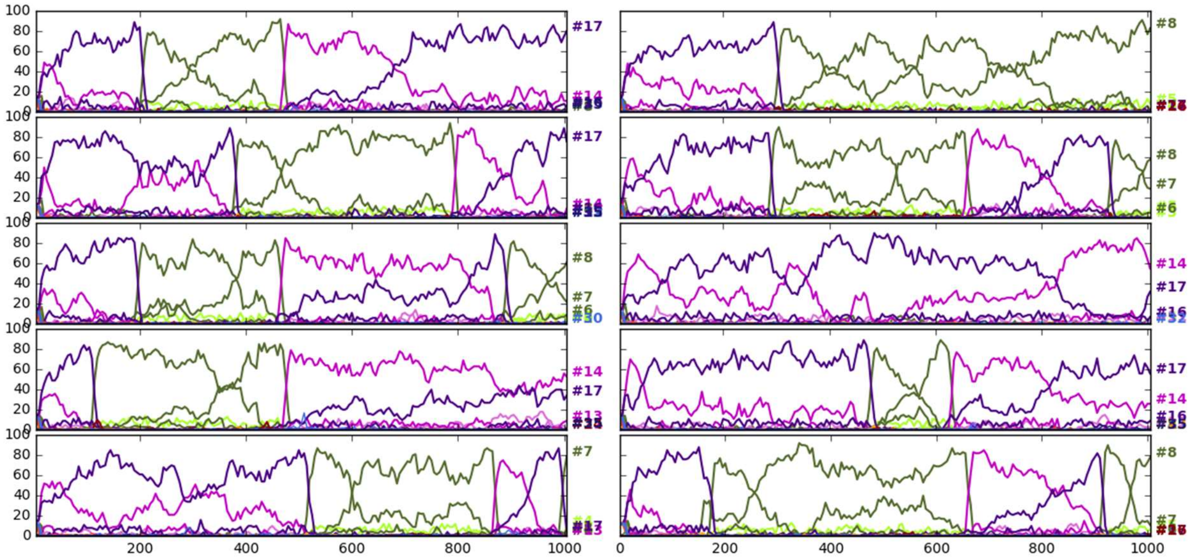

(b)

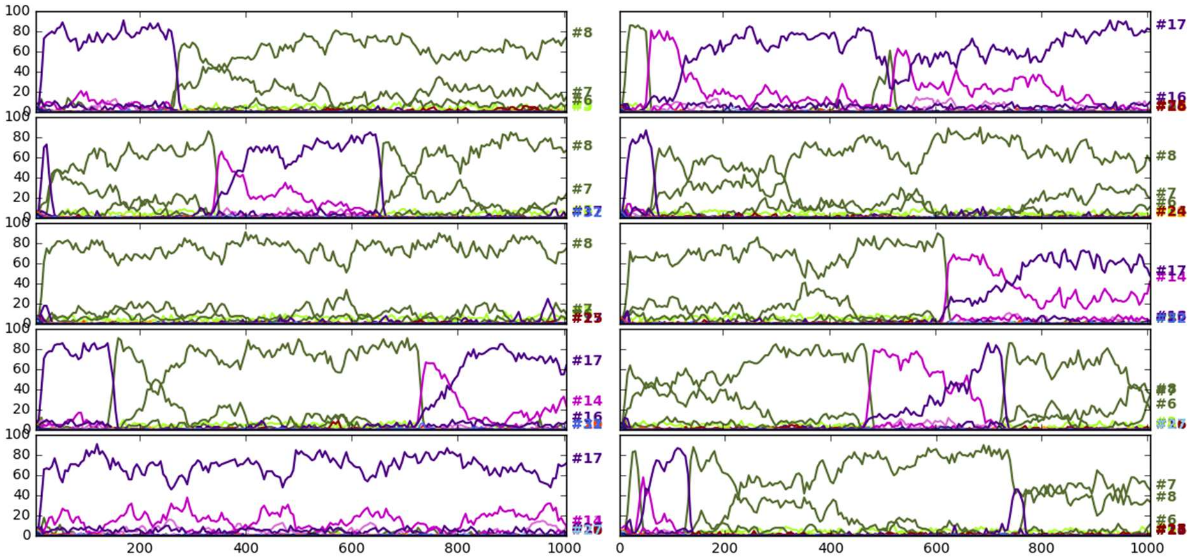

Figure 12. Timelines of the parameter combination: 3769. Pay-offs: SS09; seed: eight str.; parameter region: code11; (a) 8 strategy seed, (b) 36 strategy seed. *V* = 1.0, *Css* = 35.0, *Cww* = 7.0, *Cws* = 15.0, *Csw* = 32.0, *Ff* = 0.0.

(a)

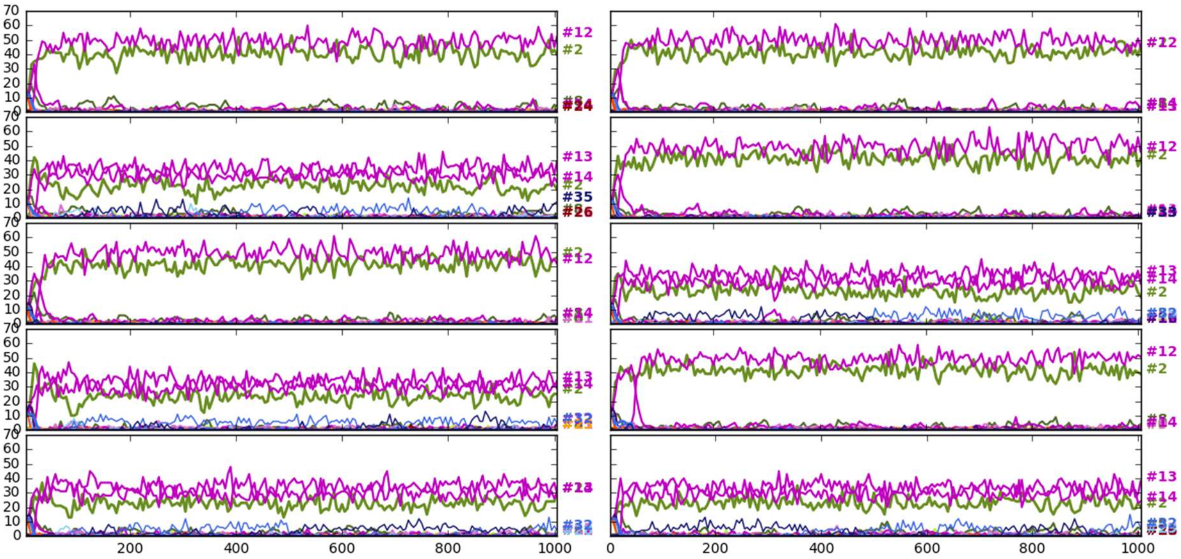

(b)

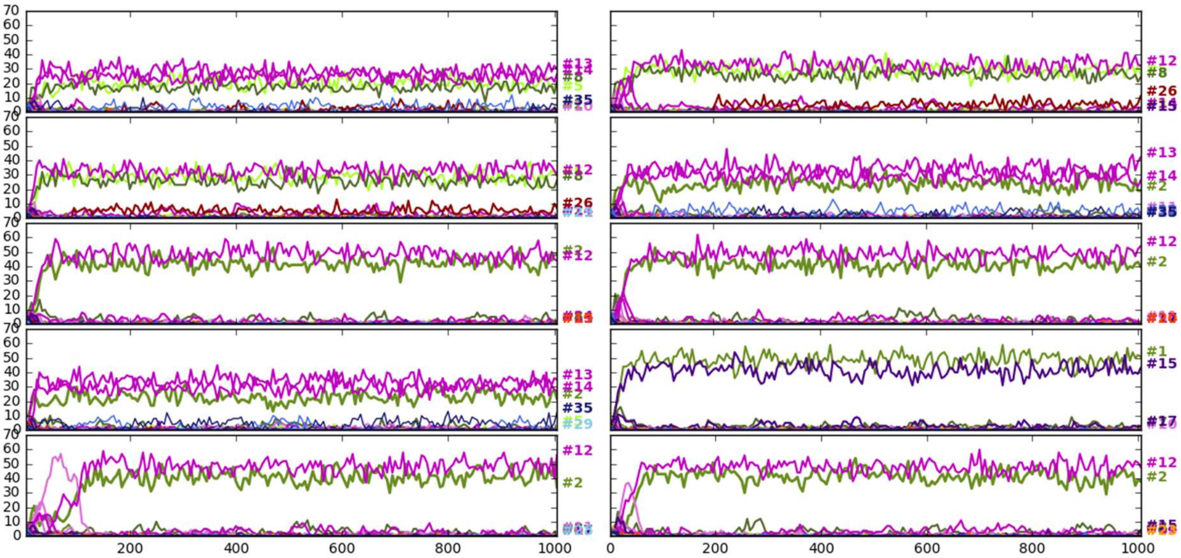

Figure 13. Timelines of the parameter combination: 2063. Pay-offs: H13; seed: eight str.; parameter region: code13; (a) 8 strategy seed, (b) 36 strategy seed. V = 19.0, Css = 25.0, Cww = 22.0, Cws = 25.0, Csw = 22.0, Ff = 18.0.

(a)

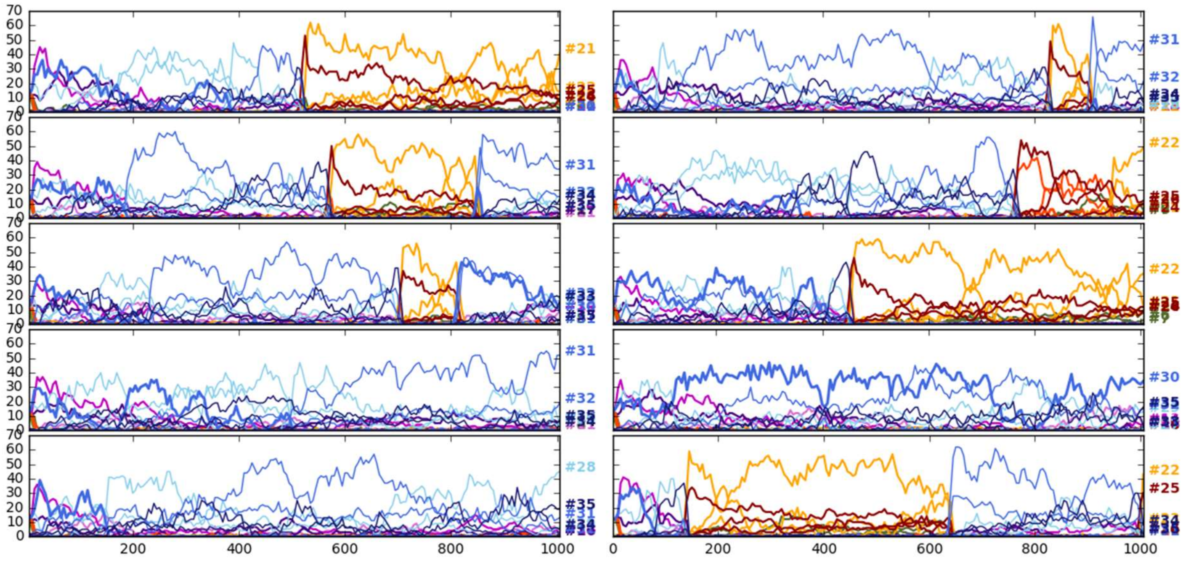

(b)

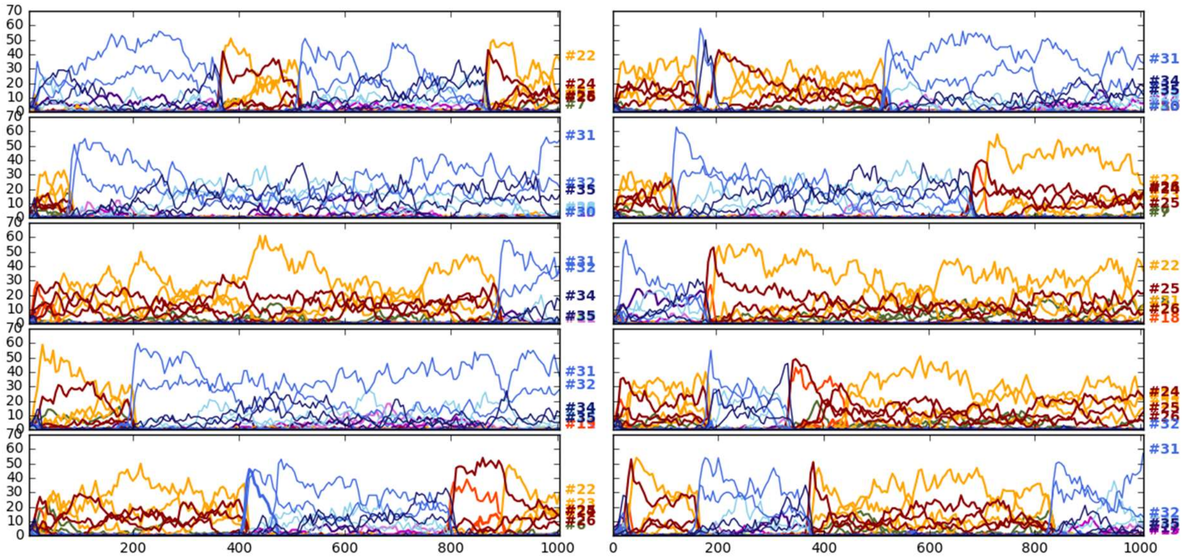

Figure 14. Timelines of the parameter combination: 2063. Pay-offs: SS09; seed: eight str.; parameter region: code13; (a) 8 strategy seed, (b) 36 strategy seed. *V* = 19.0, *Css* = 25.0, *Cww* = 22.0, *Cws* = 25.0, *Csw* = 22.0, *Ff* = 18.0.

(a)

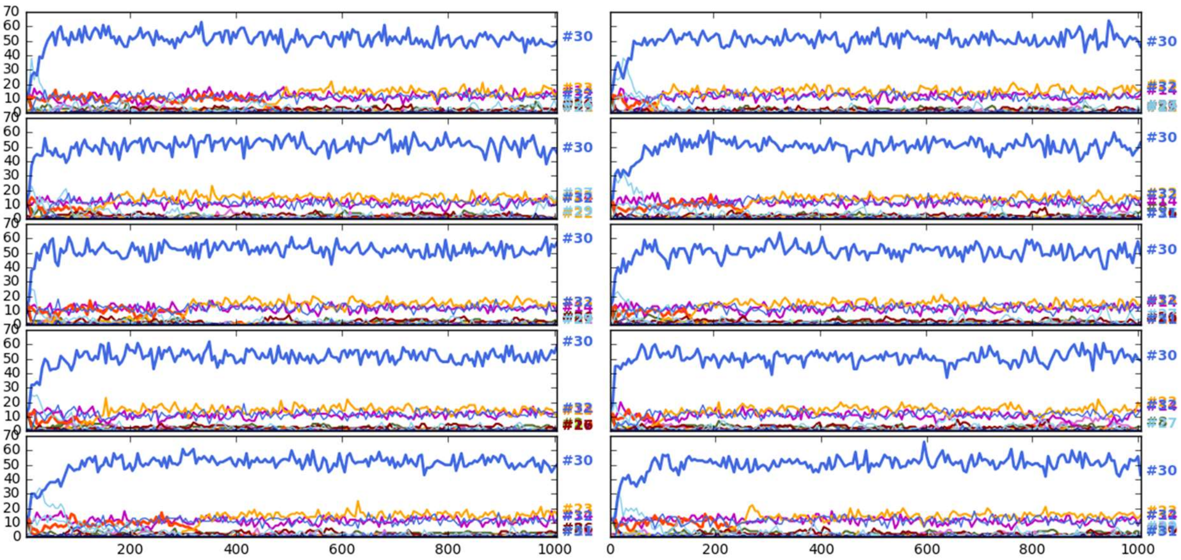

(b)

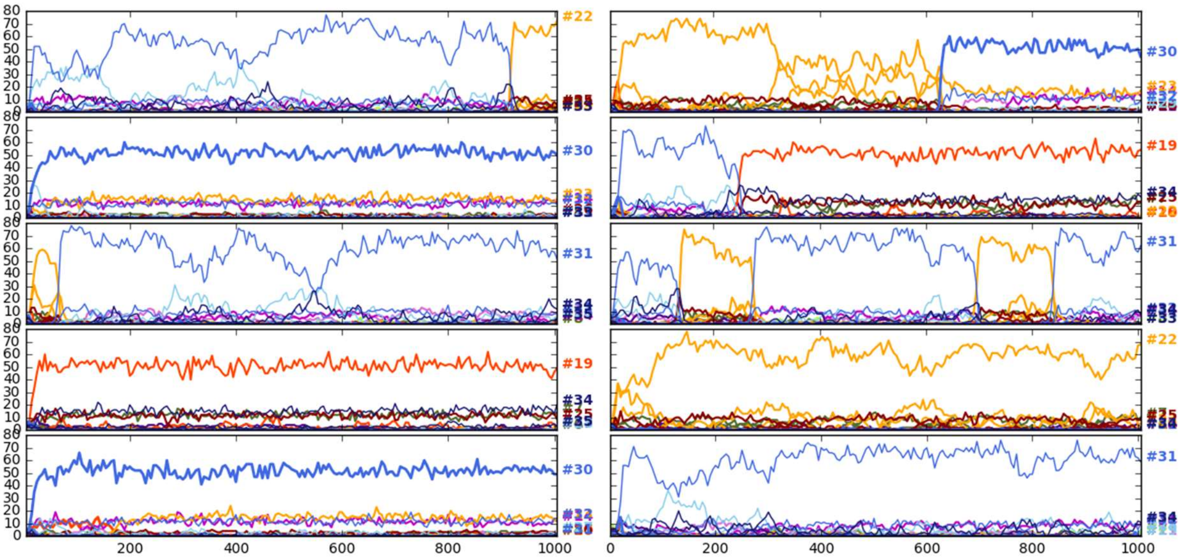

Figure 15. Timelines of the parameter combination: 677. Pay-offs: H13; seed: eight str.; parameter region: code13; (a) 8 strategy seed, (b) 36 strategy seed. *V* = 10.0, *Css* = 35.0, *Cww* = 22.0, *Cws* = 30.0, *Csw* = 22.0, *Ff* = 3.0.

(a)

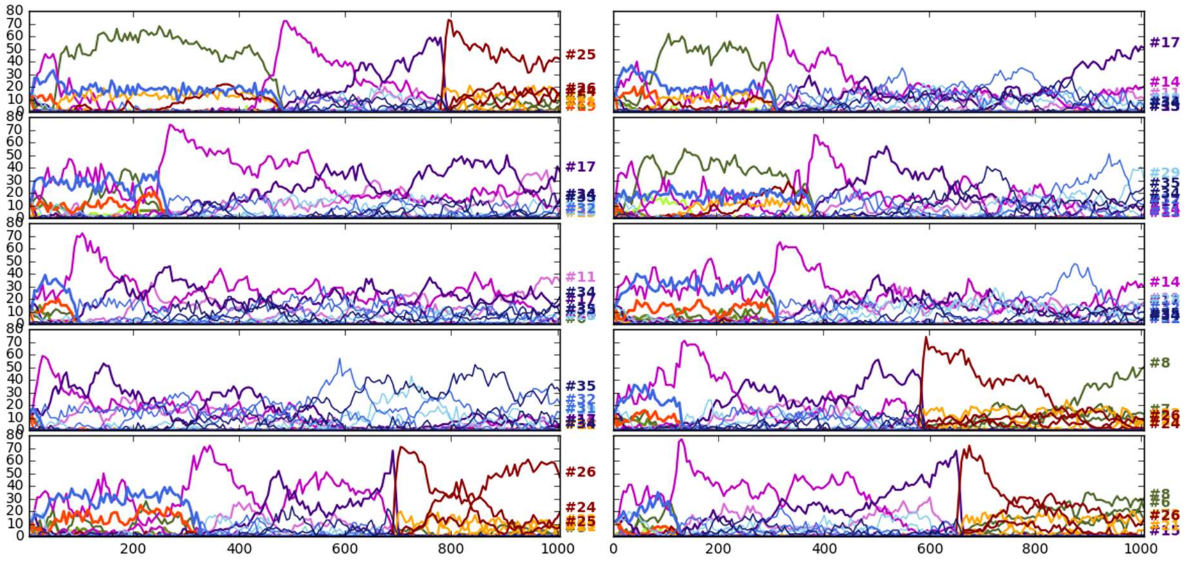

(b)

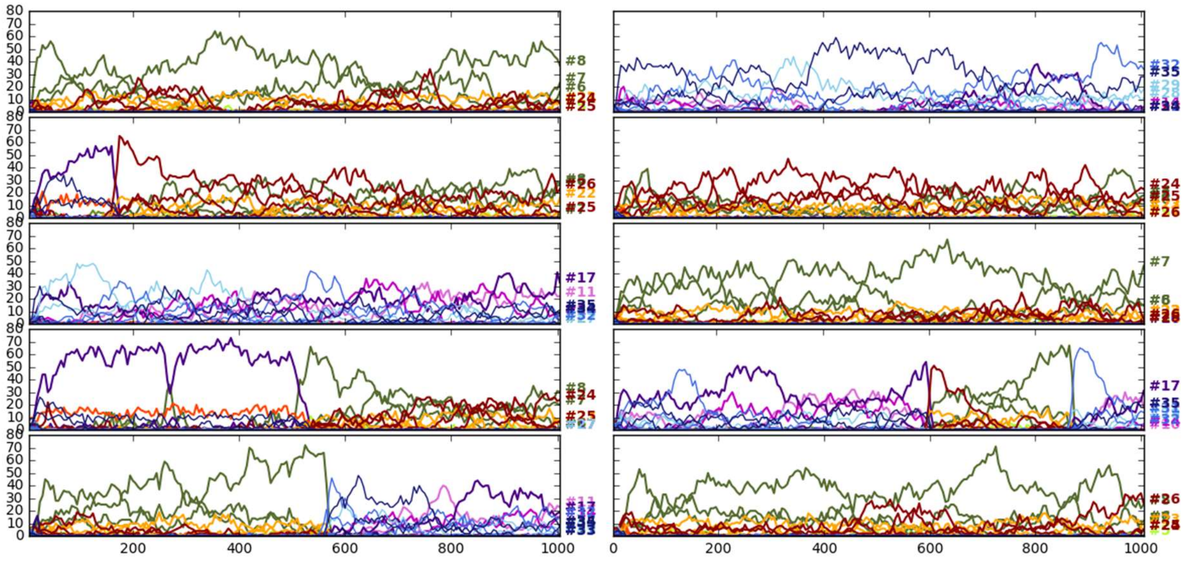

Figure 16. Timelines of the parameter combination: 677. Pay-offs: SS09; seed: eight str.; parameter region: code13; (a) 8 strategy seed, (b) 36 strategy seed. V = 10.0, Css = 35.0, Cww = 22.0, Cws = 30.0, Csw = 22.0, Ff = 3.0.

(a)

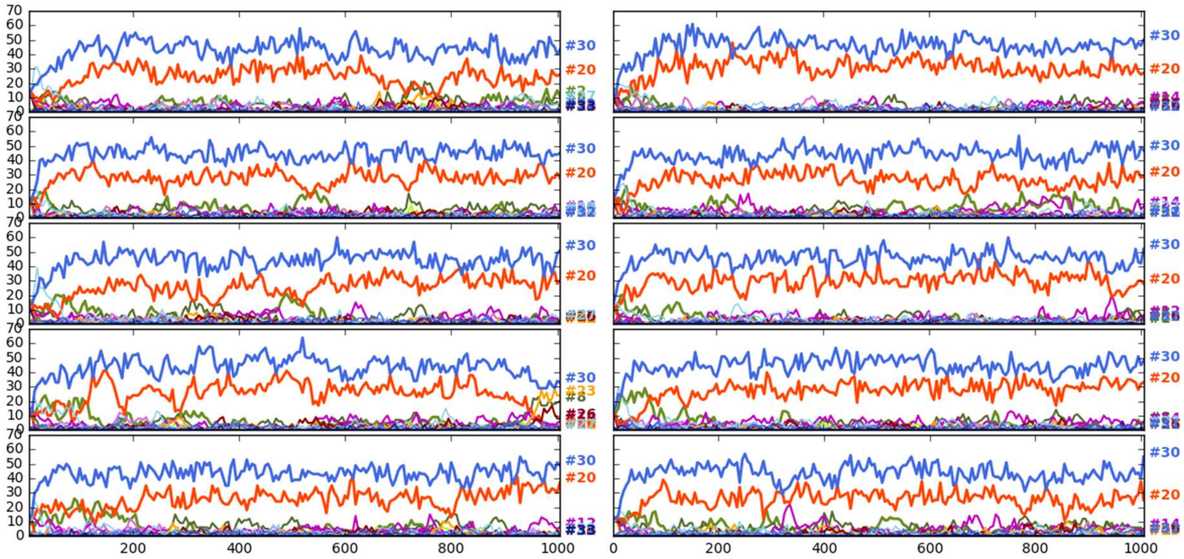

(b)

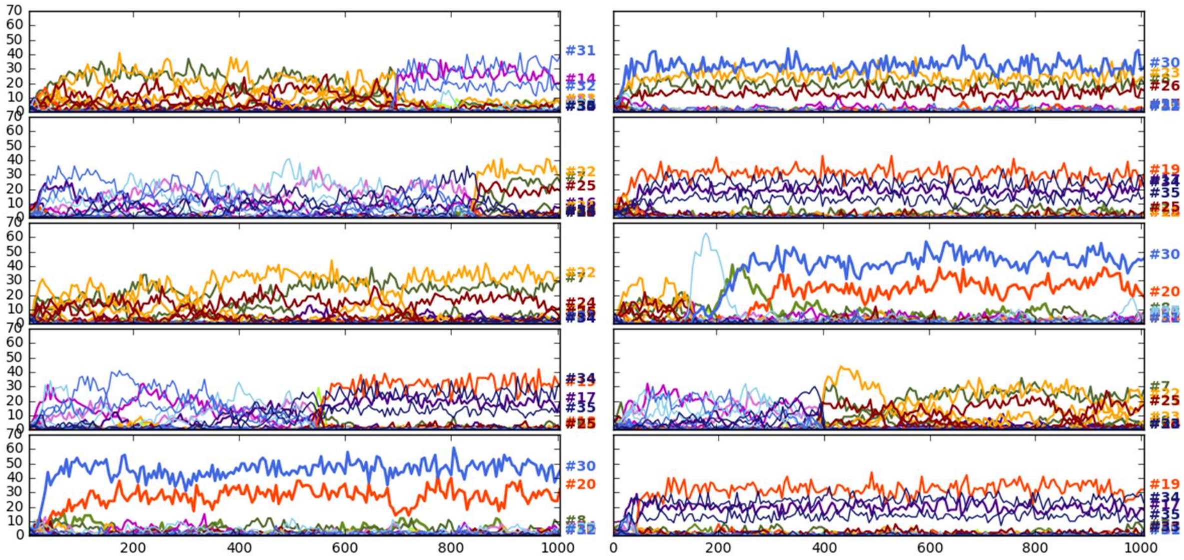

Figure 17. Timelines of the parameter combination: 183. Pay-offs: H13; seed: eight str.; parameter region: code15; (a) 8 strategy seed, (b) 36 strategy seed. *V* = 10.0, *Css* = 30.0, *Cww* = 12.0, *Cws* = 15.0, *Csw* = 12.0, *Ff* = 3.0.

(a)

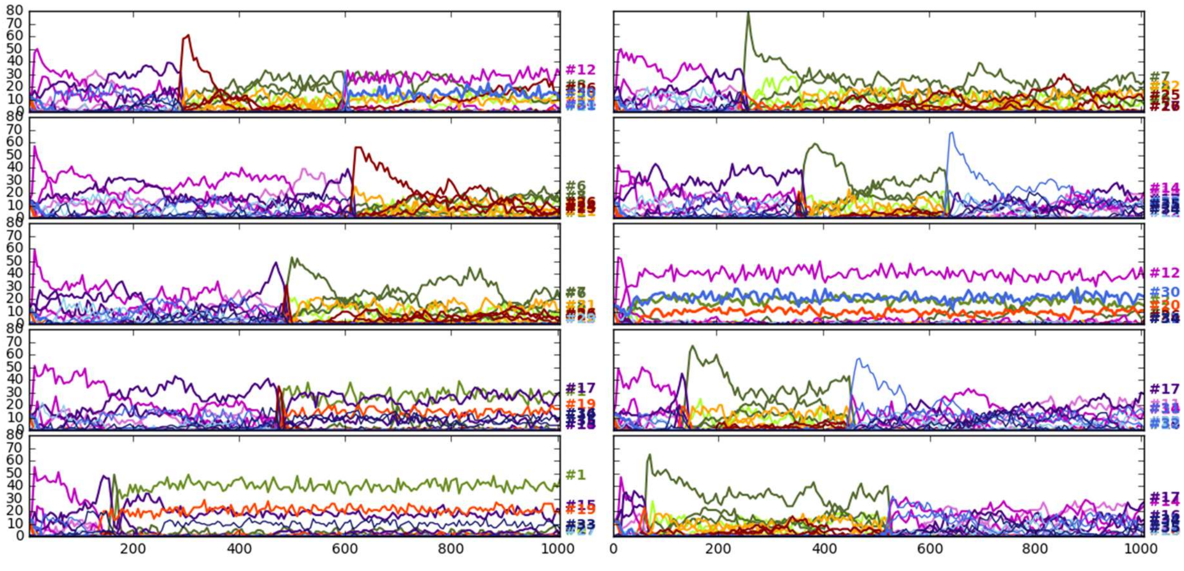

(b)

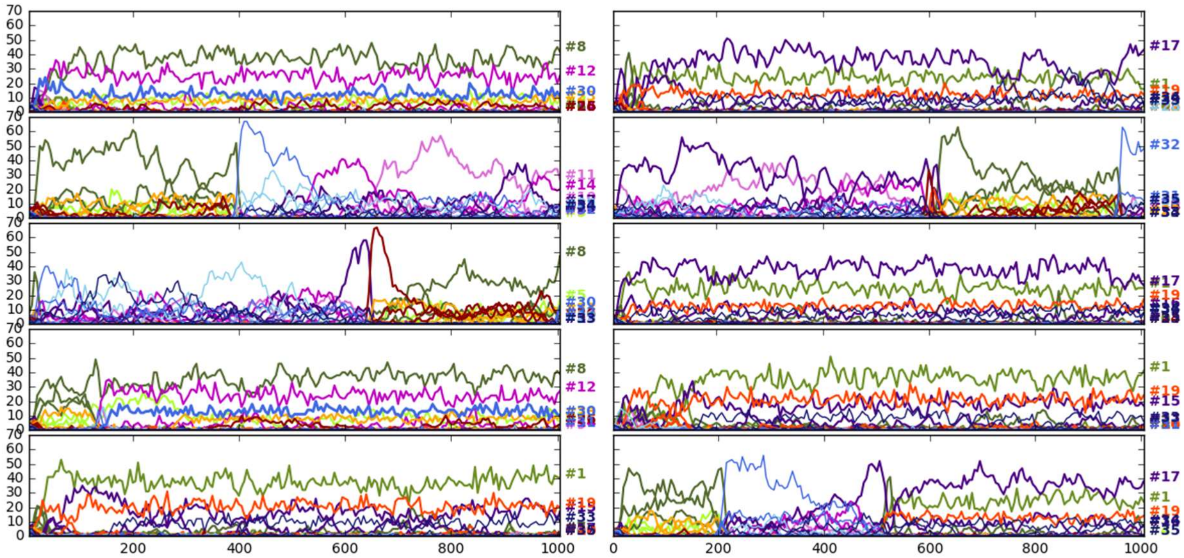

Figure 18. Timelines of the parameter combination: 183. Pay-offs: SS09; seed: eight str.; parameter region: code15; (a) 8 strategy seed, (b) 36 strategy seed. *V* = 10.0, *Css* = 30.0, *Cww* = 12.0, *Cws* = 15.0, *Csw* = 12.0, *Ff* = 3.0.

(a)

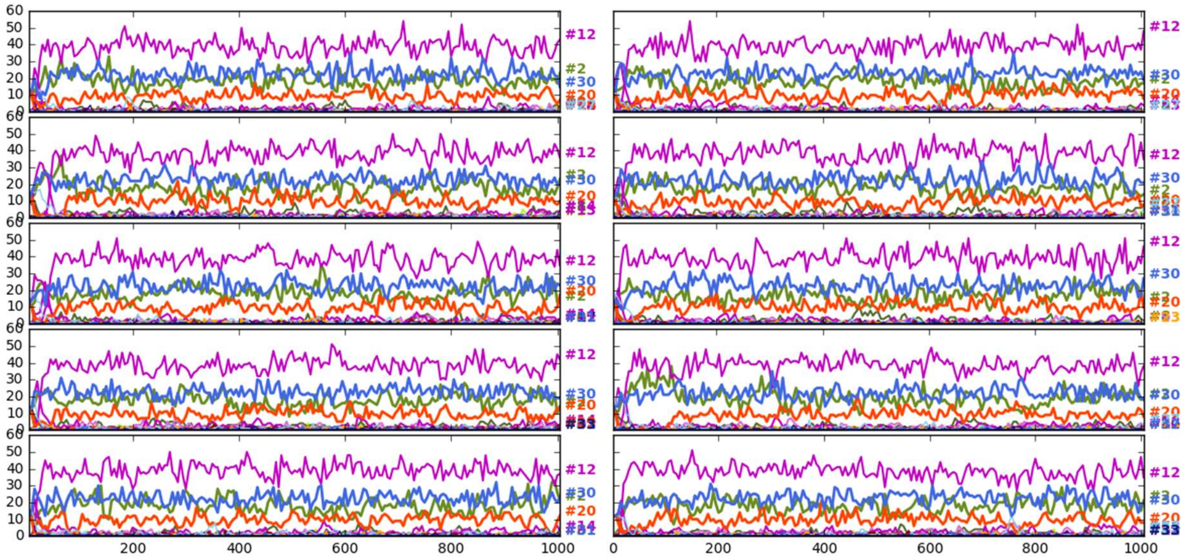

(b)

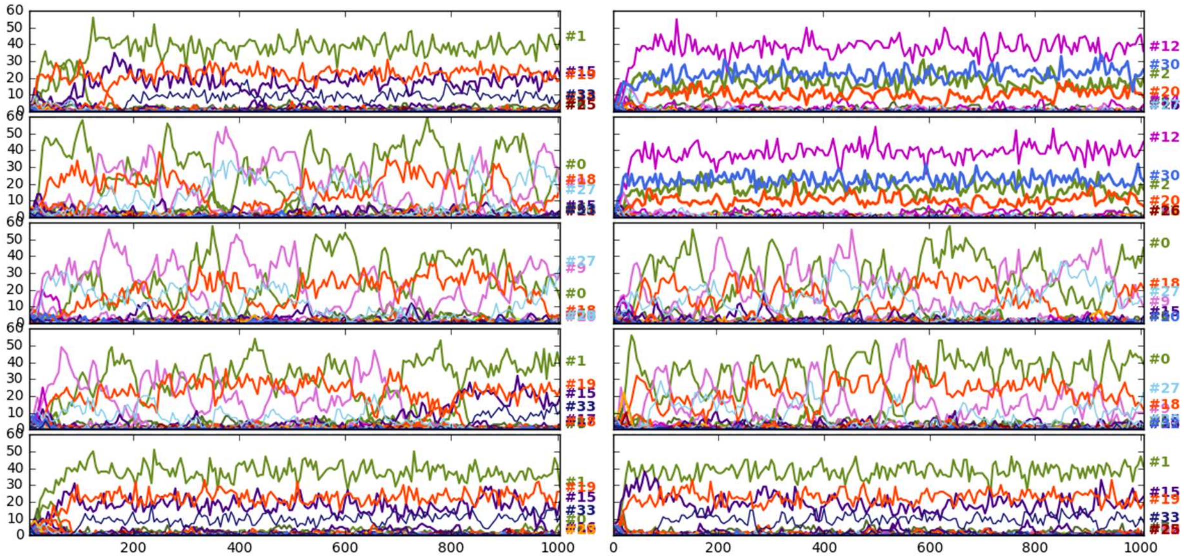

Figure 19. Timelines of the parameter combination: 4963. Pay-offs: H13; seed: eight str.; parameter region: code15; (a) 8 strategy seed, (b) 36 strategy seed. *V* = 28.0, *Css* = 35.0, *Cww* = 12.0, *Cws* = 15.0, *Csw* = 17.0, *Ff* = 0.0.

(a)

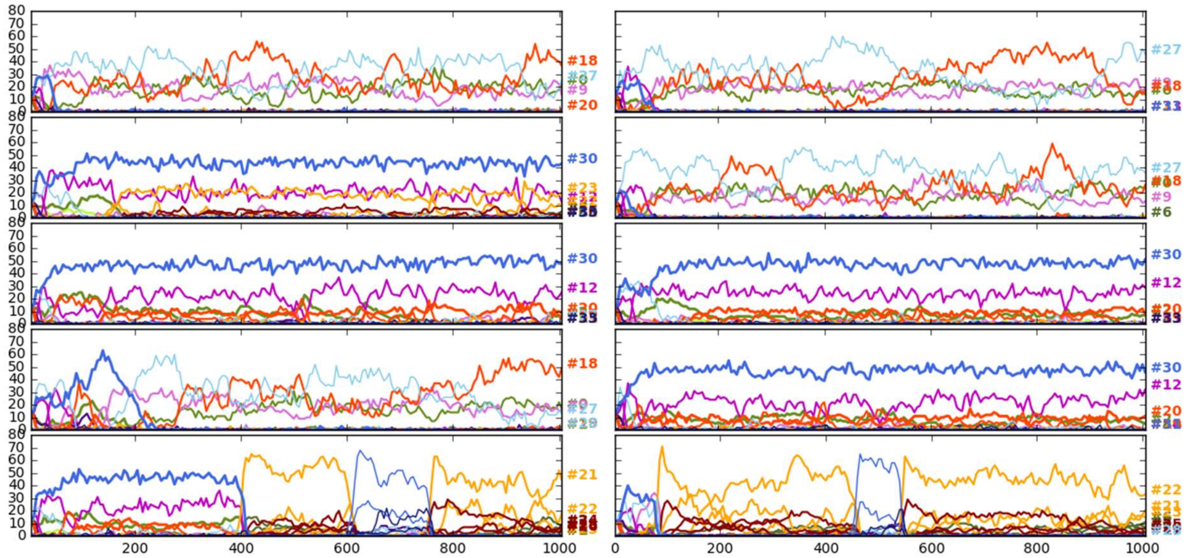

(b)

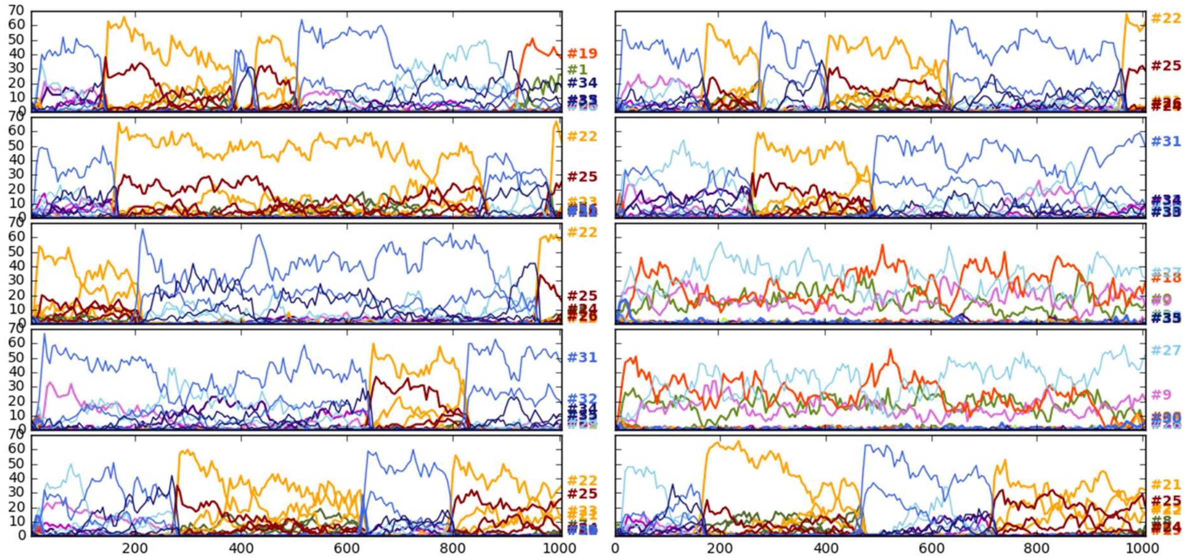

Figure 20. Timelines of the parameter combination: 4963. Pay-offs: SS09; seed: eight str.; parameter region: code15; (a) 8 strategy seed, (b) 36 strategy seed. *V* = 28.0, *Css* = 35.0, *Cww* = 12.0, *Cws* = 15.0, *Csw* = 17.0, *Ff* = 0.0.

(a)

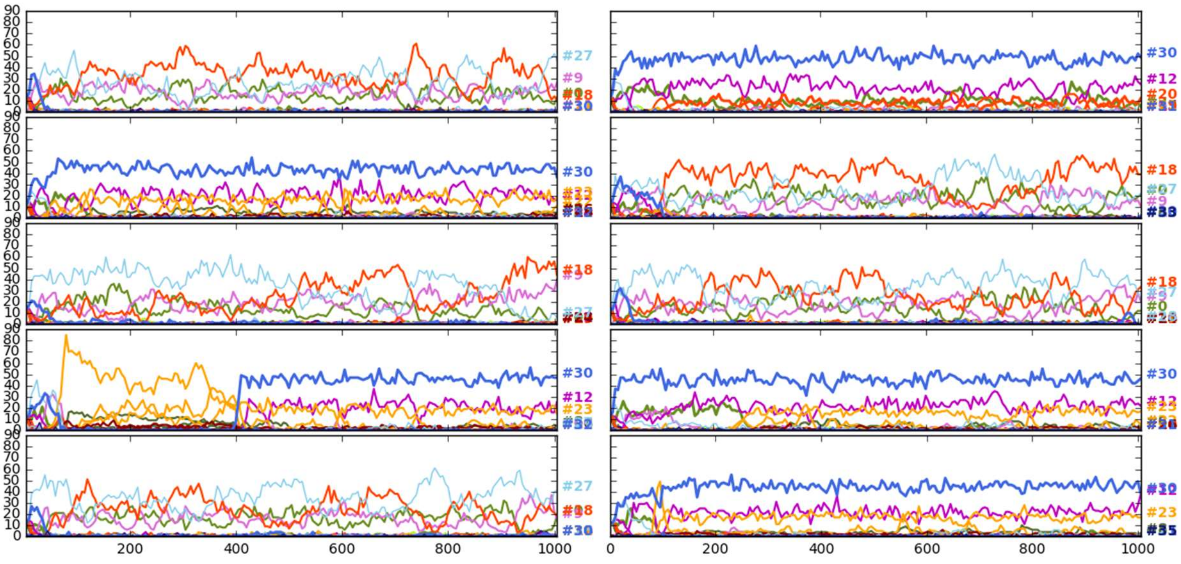

(b)

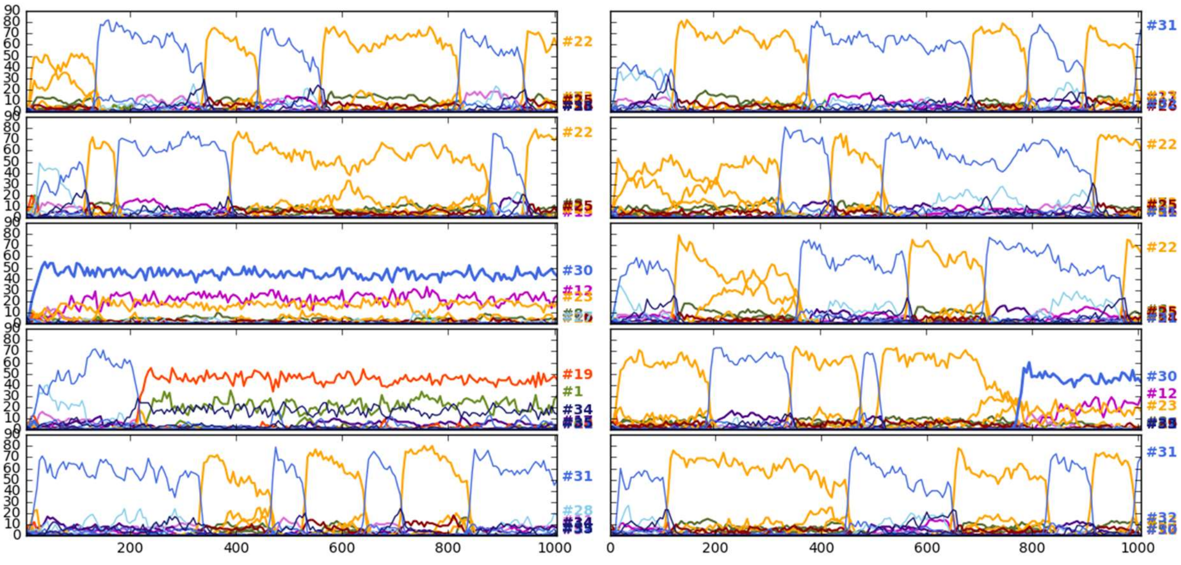

Figure 21. Timelines of the parameter combination: 3828. Pay-offs: H13; seed: eight str.; parameter region: code27; (a) 8 strategy seed, (b) 36 strategy seed. *V* = 1.0, *Css* = 35.0, *Cww* = 7.0, *Cws* = 30.0, *Csw* = 7.0, *Ff* = 6.0.

(a)

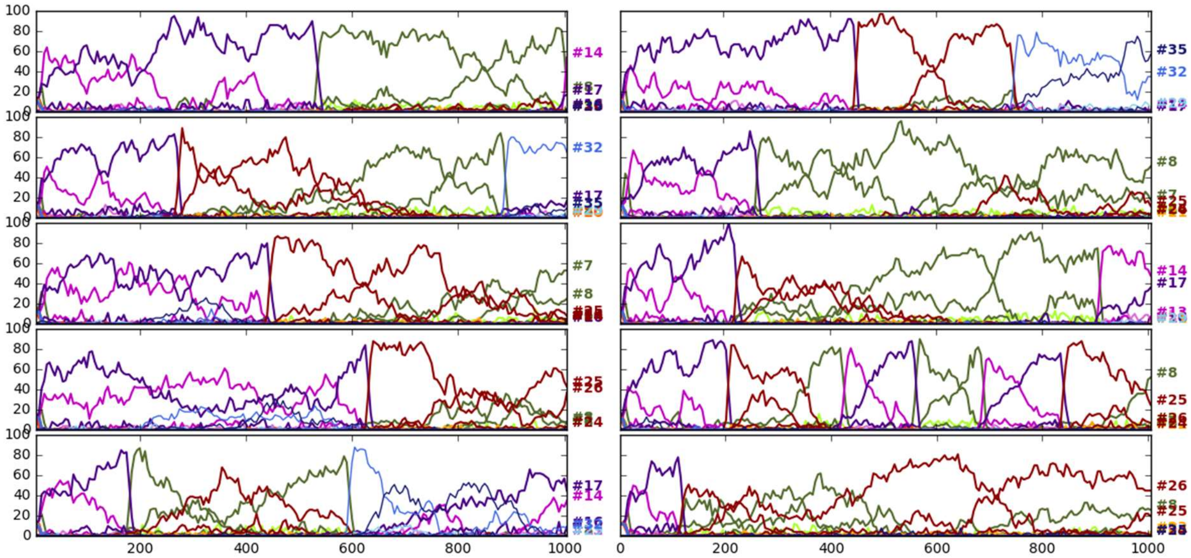

(b)

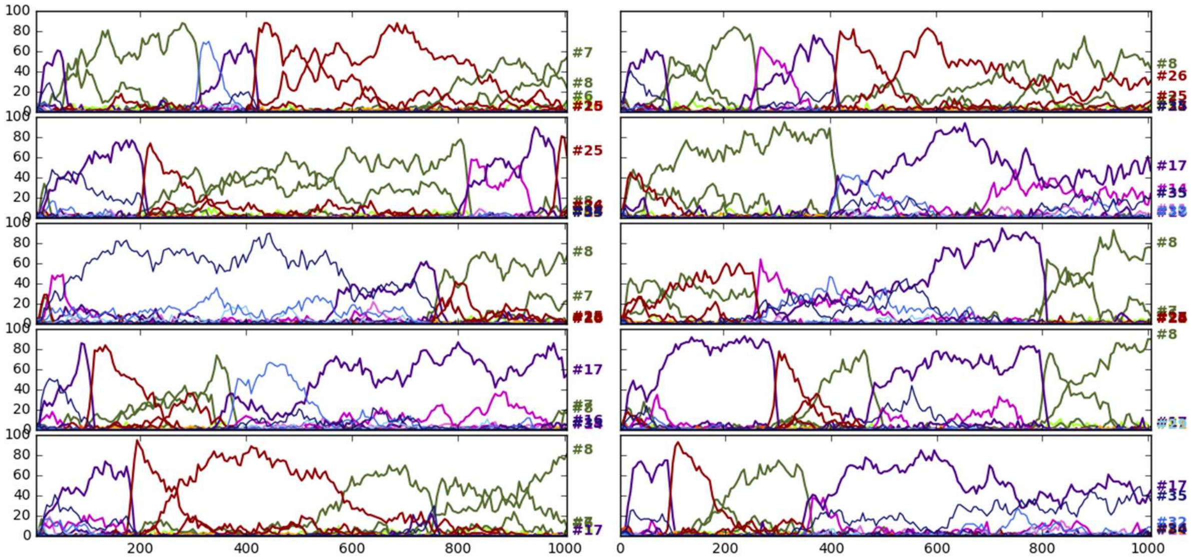

Figure 22. Timelines of the parameter combination: 3828. Pay-offs: H13; seed: eight str.; parameter region: code27; (a) 8 strategy seed, (b) 36 strategy seed. V = 1.0, Css = 35.0, Cww = 7.0, Cws = 30.0, Csw = 7.0, Ff = 6.0.

(a)

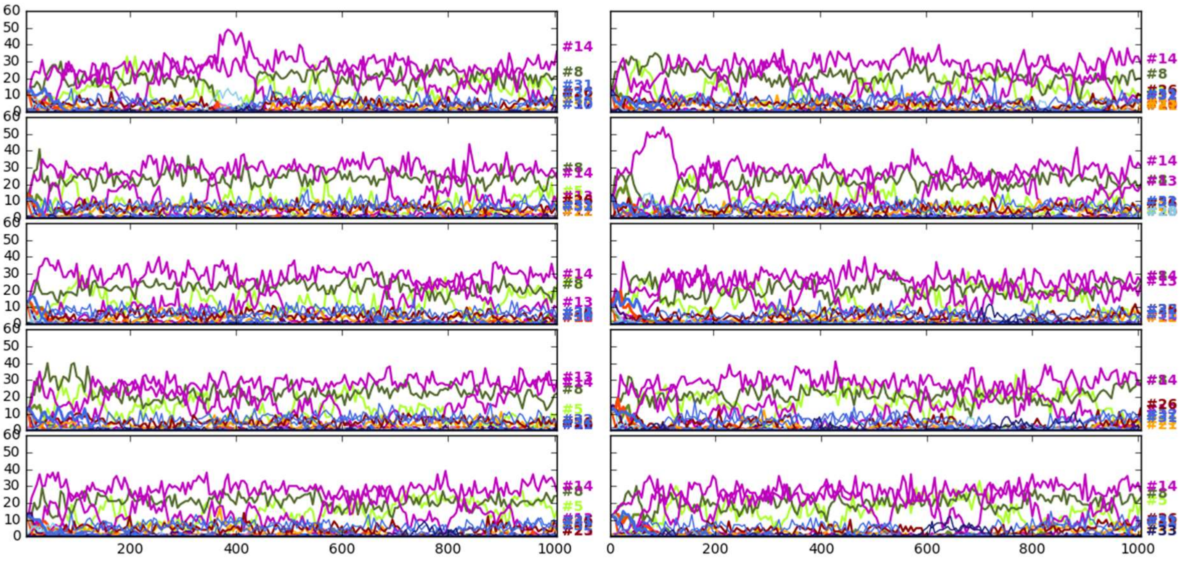

(b)

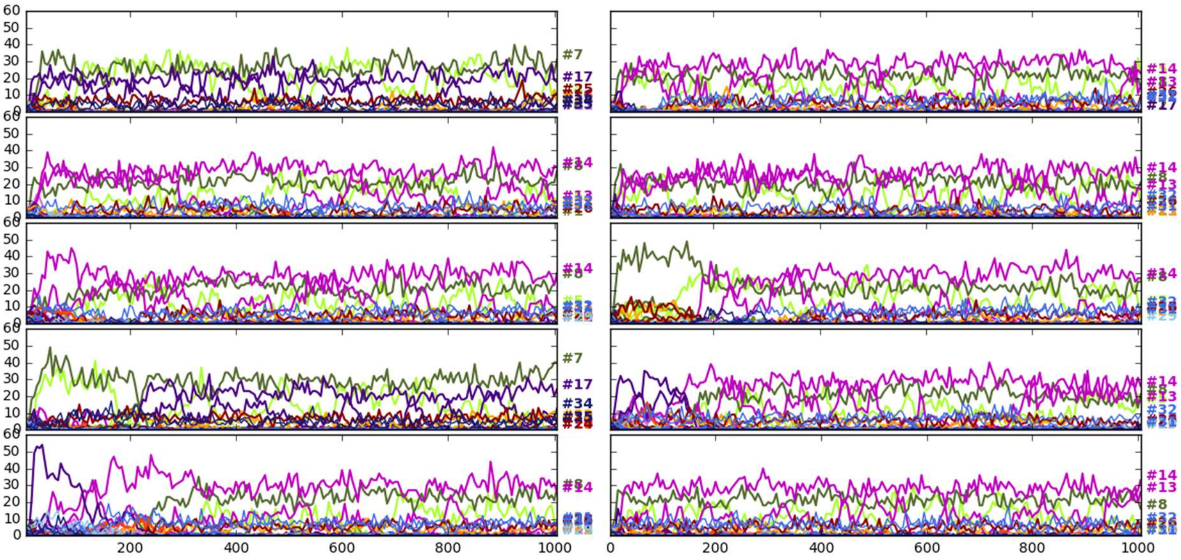

Figure 23. Timelines of the parameter combination: 3477. Pay-offs: H13; seed: eight str.; parameter region: code27; (a) 8 strategy seed, (b) 36 strategy seed. *V* = 1.0, *Css* = 35.0, *Cww* = 17.0, *Cws* = 20.0, *Csw* = 17.0, *Ff* = 15.0.

(a)

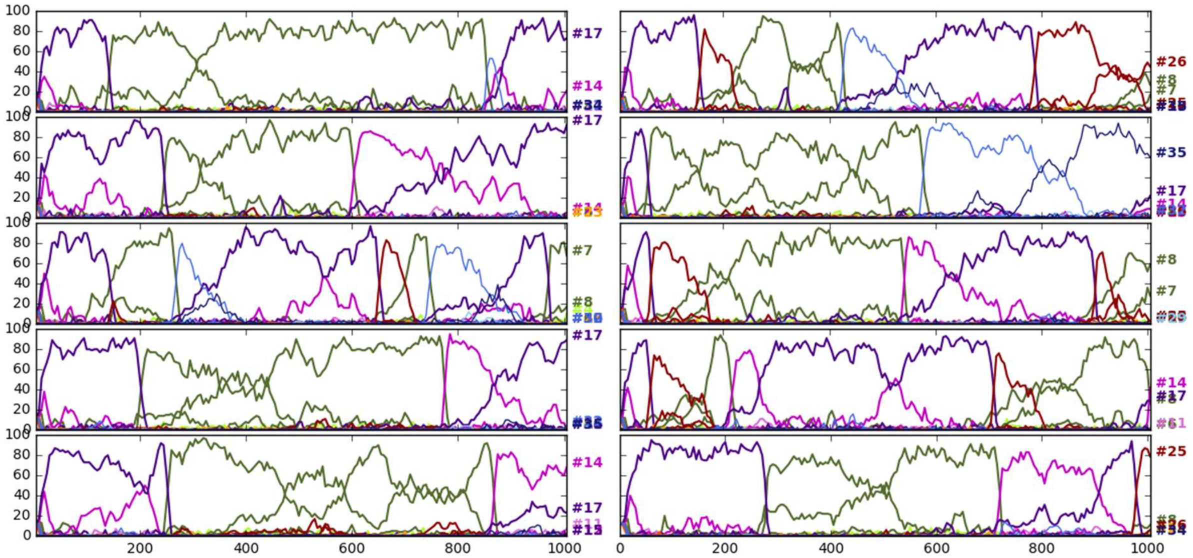

(b)

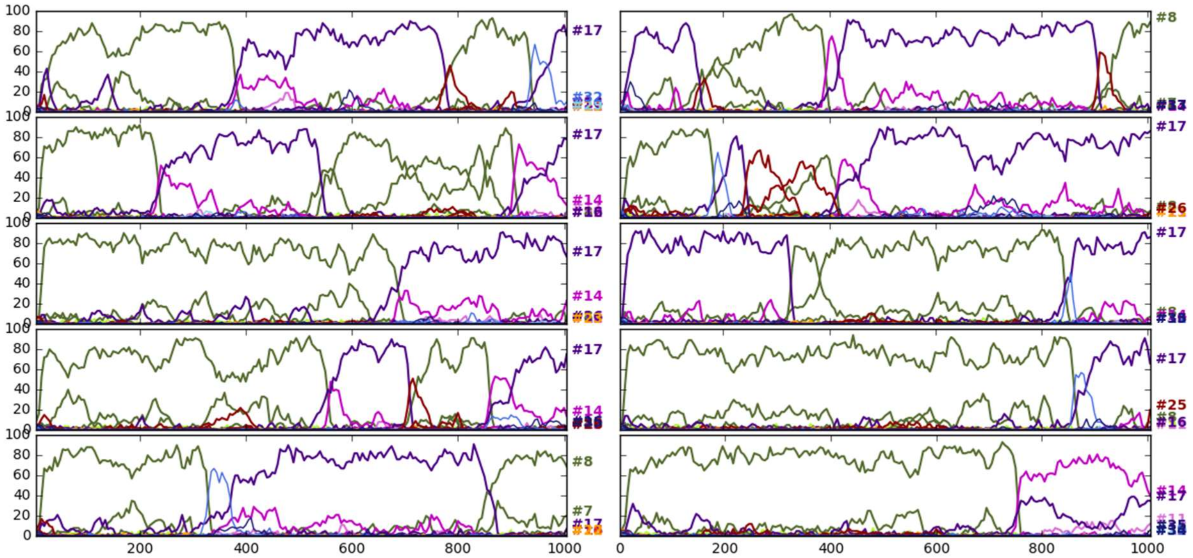

Figure 24. Timelines of the parameter combination: 3477. Pay-offs: H13; seed: eight str.; parameter region: code27; (a) 8 strategy seed, (b) 36 strategy seed. *V* = 1.0, *Css* = 35.0, *Cww* = 17.0, *Cws* = 20.0, *Csw* = 17.0, *Ff* = 15.0.

(a)

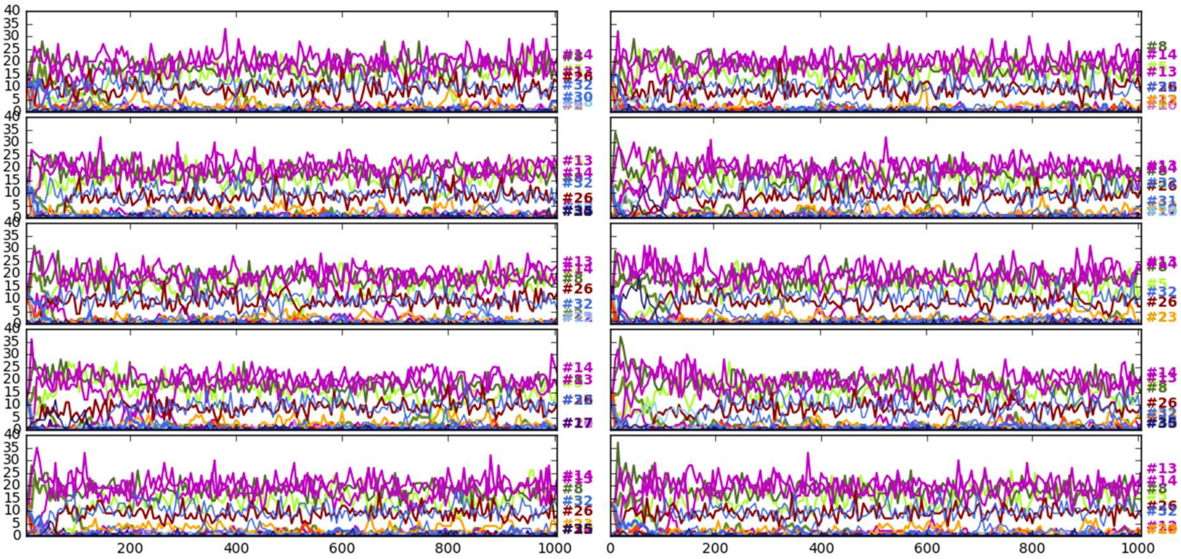

(b)

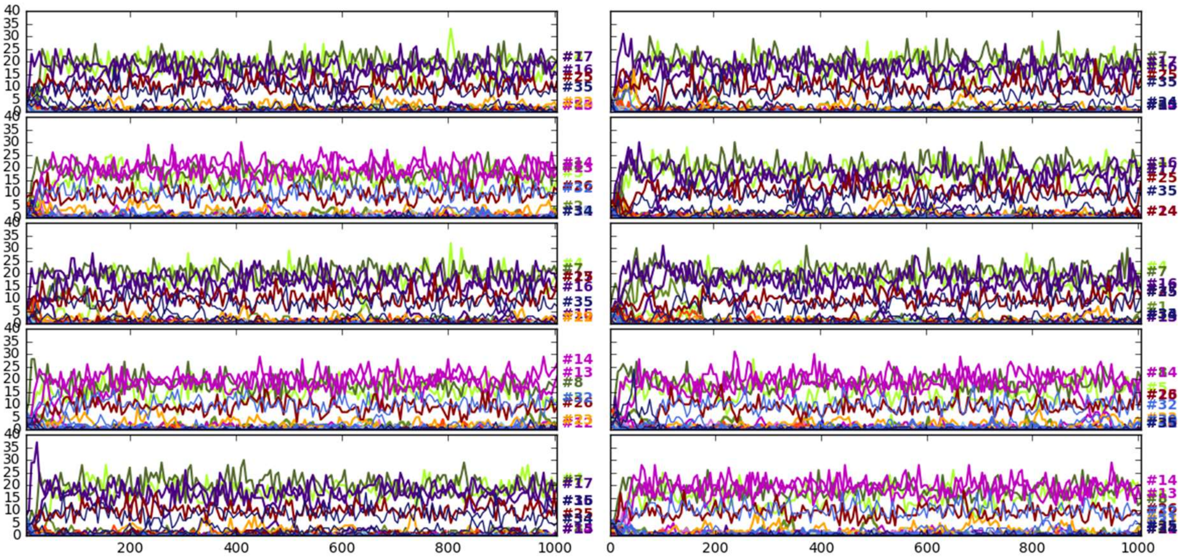

Supplement: Supplementary file 6 — Individual timelines H13 parameter set. (PDF 11297 kb) [file 12862_2017_1112_MOESM6_ESM.pdf]
